# Population genetic structure of *Oryza sativa* in East and Southeast Asia and the discovery of elite alleles for grain traits

**DOI:** 10.1038/srep11254

**Published:** 2015-06-10

**Authors:** Xiaojing Dang, Thu Giang Tran Thi, Wisdom Mawuli Edzesi, Lijun Liang, Qiangming Liu, Erbao Liu, Yang Wang, Sheng Qiang, Linglong Liu, Delin Hong

**Affiliations:** 1 State Key Laboratory of Crop Genetics and Germplasm Enhancement, Nanjing Agricultural University, Nanjing, 210095, China; 2 College of Agronomy, Hue University of Agriculture and Forestry, Hue University,102 Phung Hung Street, Hue City, Vietnam; 3 Department of Agricultural Resource and Environment, Heilongjiang University, Harbin, 150080, China

## Abstract

We investigated the nuclear simple sequence repeat (SSR) genotypes of 532 rice (*Oryza sativa* L.) accessions collected from East and Southeast Asia and detected abundant genetic diversity within the population. We identified 6 subpopulations and found a tendency towards directional evolution in *O. sativa* from low to high latitudes, with levels of linkage disequilibrium (LD) in the 6 subpopulations ranging from 10 to 30 cM. We then investigated the phenotypic data for grain length, grain width, grain thickness and 1,000-grain weight over 4 years. Using a genome-wide association analysis, we identified 17 marker-trait associations involving 14 SSR markers on 12 chromosome arms, and 8 of the 17 associations were novel. The elite alleles were mined based on the phenotypic effects of the detected quantitative trait loci (QTLs). These elite alleles could be used to improve target traits through optimal cross designs, with the expected results obtained by pyramiding or substituting the elite alleles per QTL (independent of possible epistatic effects). Together, these results provide an in-depth understanding of the genetic diversity pattern among rice-grain traits across a broad geographic scale, which has potential use in future research work, including studies related to germplasm conservation and molecular breeding by design.

Rice is a staple food for more than half of the world’s population, and two rice species are cultivated worldwide: *Oryza sativa* and *Oryza glaberrima*. The latter species, *O. glaberrima*, is cultivated only in western Africa and accounts for 5% of the global rice cultivation area. The Asian-cultivated rice species *O. sativa* is widely planted and accounts for 95% of the global rice cultivation area. There are two subspecies within *O. sativa*: *indica* and *japonica* (or *sinica*). Although the debate on the origins of *indica* and *japonica* subspecies remains contentious (single evolutionary origin^1^ versus multiple origin^2^), the separate distributions of the subspecies, with *indica* distributed at low latitudes (from 34°S to 33°N) in warm climate districts and *japonica* distributed at high latitudes (higher than 33°N or 34°S) in cool climate districts, is indisputable. Knowledge of the spatial genetic structure of these two subspecies in natural populations is important for understanding the clinal variations and improving the cultivars by mining elite alleles. With the decreasing farmland area, increasing global population and changing climate, there is an urgent need to ensure grain production^3^.

The grain number, panicle number, and grain weight are three important components of the grain yield of rice (*O. sativa* L.). When the panicle number per unit area and grain number per panicle reach an ideal level, the improvement of grain weight plays a key role in further increases in yield in rice-breeding programs^4^. Grain weight is determined largely by the grain size, which mainly includes the grain length (GL), grain width (GW) and grain thickness (GT). In addition, GL, GW and GT are important parameters for evaluating the appearance of rice, which directly relates to the trade value of rice.

Grain length, width and thickness and 1,000-grain weight are quantitative traits controlled by multiple genes. To date, many quantitative trait locus (QTL) studies of grain traits have been reported based on bi-parental family populations. The detected QTLs that control GL, GW and TGW were distributed in all twelve chromosomes^5^,^6^,^7^,^8^,^9^,^10^,^11^,^12^,^13^,^14^,^15^, whereas the detected QTLs that control GT were located on chromosomes 3, 5, 6, 9, 10 and 11^14^,^15^. Recently, several genes have been shown to control grain size, including *GS3*^16^,^17^, *DEP1*^18^ and *GL3*^19^, which regulate grain length and grain weight, and *GW2*^20^ (encoding a RING-type E3 ubiquitin ligase), *qSW5*^21^, *GS5*^22^ (encoding a putative serine carboxypeptidase) and *GW8*^23^ (encoding a transcription factor with squamosa promoter binding protein-like (SBP) domain), which regulate grain width. Despite the success of QTL analyses, the major limitation of linkage mapping is that only two alleles at any given locus can be studied in biparental crosses of inbred lines.

Association mapping based on linkage disequilibrium (LD) has recently emerged as an alternative approach to detecting QTLs by taking into account marker-trait associations, and it enables researchers to exploit the natural diversity of the genome and locate valuable genes^24^. Association mapping has been widely applied to the mining of excellent alleles in humans^25^; animals such as chickadees (*Poecile atricapillus*)^26^ and ptarmigan (*Lagopus mutus*)^27^, and plants, such as rice (*O. sativa*)^28^,^29^,^30^, maize (*Zea mays*)^31^, wheat (*Triticum aestivum*)^32^, soybean (*Glycine max*)^33^ and foxtail (*Setaria viridis*)^34^.

Recent many studies have reported the discovery of elite alleles for grain traits in rice by using single nucleotide polymorphism (SNP) markers in association analyses. These studies found 19 significant associated loci for grain length, which were distributed on chromosomes 1, 3, 4, 5, 6, 7 and 12; 16 significant associated loci for grain width, which were distributed on chromosomes 2, 5, 7, 8, 10, 11 and 12; and 7 significant associated loci for grain weight, which were distributed on chromosomes 1, 2, 4, 7 and 8^28^,^35^,^36^. However, the SNPs showed a high false positive rate^34^,^35^. Simple sequence repeat (SSR) loci are particularly useful for the study of population structure because their high level of allelic diversity facilitates the detection of the fine diversity more efficiently than an equal number of restriction fragment length polymorphisms (RFLPs), amplified fragment-length polymorphisms (AFLPs), or SNP loci^37^.

The objectives of this study are to (1) evaluate the population structure and genetic diversity of Asian rice, (2) detect the extent of LD between pairs of SSR markers in an entire rice genome, (3) detect the QTLs controlling grain traits and mine elite alleles and (4) explore the optimal cross design for cultivar improvement using population genetic analyses and association mapping with a set of 532 rice accessions using SSRs in four environments.

## Results

### Genetic diversity of SSR markers

All 258 SSR markers were polymorphic, and they produced a total of 2698 alleles among the 532 assayed accessions. The proportion of rare alleles (frequency less than 5%) within the 2698 identified alleles was 36.25%. The average number of alleles per locus was 10.46, with values ranging from 2 (RM437_Chr 5 and RM7163_Chr 11) to 25 (RM7545_Chr 10) (Supplementary Table 1). The average genetic diversity over all SSR loci was 0.7320, with values ranging from 0.0765 (RM7163_Chr 11) to 0.9424 (RM7545_Chr 10) (Supplementary Table 1). The mean polymorphism information content (PIC) value was 0.7042, with values ranging from 0.0736 (RM7163_Chr 11) to 0.9394 (RM7545_Chr 10) and a major distribution between 0.5167 and 0.9008 (Supplementary Table 1). Two hundred and twenty-two markers (86.1%) were highly informative (PIC > 0.5), 29 (11.2%) were moderately informative (0.5 > PIC > 0.25) and 7 (2.7%) were slightly informative (PIC < 0.25).

### Population structure and genetic relatedness

**Figure 1:**
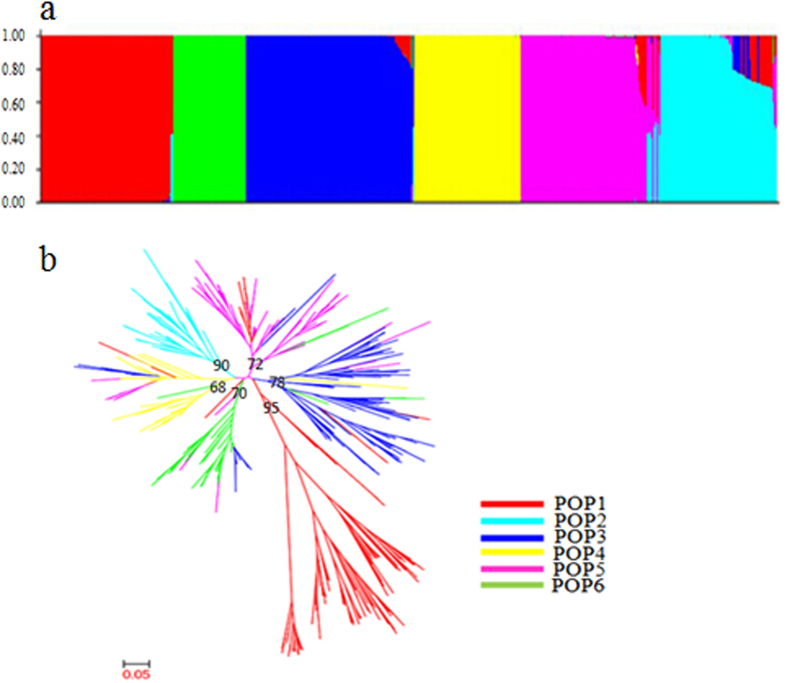
Structure analysis of 532 rice accessions using **a**: STRUCTURE; **b**: Unrooted neighbor-joining. a Posterior probability of each rice variety of 532 rice accessions belonging to six subpopulations calculated by STRUCTURE software. Each accession is represented by a vertical bar. The colored subsections within each vertical bar indicate membership coefficient (Q) of the accession to different clusters. Identified subpopulations are POP1 (red), POP2 (green), POP3 (navy blue), POP4 (yellow), POP5 (purple), POP6 (light blue). **b** Dendrogram of 532 rice accessions by unweighted neighbour-joining of simple matching coefficients based on SSR genotyping data. Identified subpopulations are POP1 (red), POP2 (light blue), POP3 (navy blue), POP4 (yellow), POP5 (purple), POP6 (green). Bootstrap values (out of 100) are identified at the branch points.

An analysis of the model-based population structure provided evidence of a significant population structure in the 532 rice accessions and identified the highest likelihood value at K = 6 for all five replicates (five runs for each K) (Fig. 1a, Supplementary Fig. 1). The population structure data based on the Q matrix for each accession are summarized in Supplementary Table 2. A neighbour-joining tree of the 532 accessions was constructed based on Nei’s genetic distance (Fig. 1b), and the results were consistent with the results from the Structure analysis.

The non-admixed accessions in each subpopulation were determined using the Q-matrix assignment of above 0.9. The number of non-admixed accessions from POP1 to POP6 was 94, 54, 147, 65, 69 and 45. The 94 accessions in POP1 were all from Vietnam; the 54 accessions in POP2 were mainly from northeastern China and Japan; the 147 accessions in POP3 represented landraces from the Taihu Lake valley; the 65 accessions in POP4 were mainly from northern China (northern Jiangsu, Anhui, Henan, and Shandong provinces and Tianjin City); the 69 accessions in POP5 were modern improved varieties mainly from the Taihu Lake valley; the 45 accessions in POP6 were mainly from South China (Taiwan, Yunnan and Hunan provinces); and the remaining 58 accessions showed admixed ancestry, and they were excluded in subsequent analyses.

Genetic relatedness analysis indicated that the accessions in this study were distantly related (Supplementary Fig. 2), with greater than 80% of the kinship coefficient values at less than 0.05, 5.6% ranging from 0.05–0.10 and the remaining 12.4% showing various degrees of genetic relatedness. This result suggests that there was weak or absent relatedness between the pairwise rice accessions. Based on the results of the relatedness analysis, a K matrix was constructed for the association analysis.

### Genetic differentiation across subpopulations

The average *F*_ST_ among the six subpopulations was 0.287, with the *F*_ST_ for each locus ranging from 0.092 for RM333_Chr 10 to 0.862 for RM4835_Chr 4. The pairwise comparison based on the values of *F*_ST_ could be interpreted as standardized population distances between the two subpopulations. The pairwise *F*_ST_ value in the present study ranged from 0.142 (between POP3 and POP4) to 0.456 (between POP1 and POP2), with an average value of 0.286 (Table 1). The results of the analysis of molecular variance (AMOVA) indicated that 28.9% of the total genetic variation occurred between the subpopulations, whereas 71.1% occurred within the subpopulations (Supplementary Table 3). These results indicate a high degree of genetic differentiation across the six subpopulations.

### Average standardized individual allele size of six subpopulations

**Figure 2:**
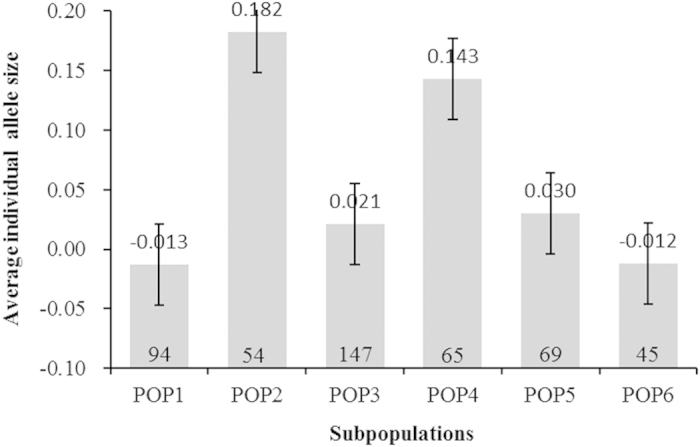
Average individual allele size for the six subpopulations (from POP1 to POP6). The mean, the standard error, and the number of accessions per subpopulation are presented.

To test whether the directional evolution of SSR size occurs in rice, we compared the average standardized individual allele size in the geographically derived groups (POP2-POP6) to that of the low-latitude Vietnam group (POP1). The results showed that the average standardized individual allele size increased with latitude. The average standardized individual allele size was smallest (−0.013) in Vietnam (POP1) and largest (0.182) in northeastern China and Japan (POP2) (Fig. 2). Highly significant differences were found between POP2 and POP1 (*t* = −5.33, *P* < 0.01), POP3 and POP1 (*t* = 6.18, *P* < 0.01), PO*P*4 and POP1 (*t* = −5.96, *P* < 0.01), and POP5 and *P*OP1 (*t* = 4.83, *P* < 0.01) (Fig. 2).

### Linkage disequilibrium

There were 22,742 significant LDs out of 33,153 pairs (based on D', *P* < 0.05), which includes both interchromosomal and intrachromosomal combinations. Among the 22,742 pairs showing significant LD, 12.1% of them were intrachromosomal combinations (2757 pairs). For the average D' values, POP1 had the lowest LD (0.419), whereas POP3 had the highest (0.474) (Supplementary Table 4).

**Figure 3:**
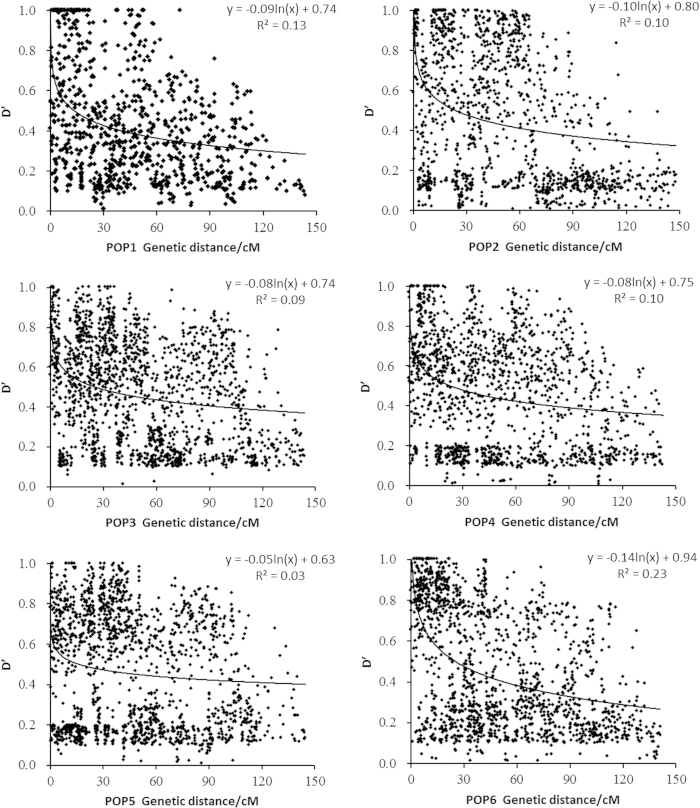
Relationship between D' value and genetic distance of syntenic marker pairs in subpopulations.

We used the D' value corresponding to intrachromosomal SSR loci as well as the genetic distance in each subpopulation to draw the attenuation map. Figure 3 shows that the D' values decay with increasing genetic distance (cM). Regression analysis between the D' values and genetic distances of the syntenic marker pairs revealed that the six subpopulation genomes fit the equation *y* = *b* ln*x* + *c*. The minimum distances of LD decay for POP1-POP6 were 13.6 cM, 23.7 cM, 25.2 cM, 23.4 cM, 16.5 cM and 25.3 cM. Apparently, POP1 had the highest decay velocity with the shortest decay distance, whereas POP3 and POP6 demonstrated the lowest decay velocity among the 6 subpopulations.

### Phenotypic distributions and correlations between multiple traits

The phenotypic data of the GW, GT and TGW traits in the studied population followed a normal distribution, whereas GL followed a skewed distribution based on the skewness values and kurtosis statistics (Table 2). The largest and smallest values for the GL, GW, GT and TGW traits over 4 years were approximately 13 and 6 mm, 4 and 2 mm, 3 and 1.7 mm and 33 and 16 g, respectively, and they had average values of 8 mm, 3 mm, 2 mm and 25 g in the population (Table 2). A two-way ANOVA showed that significant differences occurred among the tested cultivars (*P* < 0.01), indicating a large amount of genetic variation in the 532 cultivars. The average broad-sense heritability value for the GL, GW, GT and TGW traits over 4 years was 98.26%, 95.86%, 96.31% and 95.53%, respectively.

GL was correlated negatively with GW and GT but not significantly correlated with TGW, and the correlations of TGW with GW and GT and of GW with GT were significantly positive (Supplementary Table 5).

### Significant marker-trait association loci detected across the entire population

A marker-trait association analysis based on a mixed linear model (MLM) revealed that eight markers located on chromosomes 3, 4, 6, 8, 9 and 11 were associated with GL (Table 3). The range of phenotypic variation explained (PVE) was from 2.4% to 13.1%. RM335_Chr 4, which resides on 5.4 cM, had the maximum PVE for GL, which was 12.9% in 2010, 12.8% in 2011, 13.1% in 2012 and 10.8% in 2013 (Table 3). Four markers distributed on three chromosomes were associated with GW (Table 3), of which RM1019_Chr 8 had the highest PVE, with values of 15.2% in 2010, 12.1% in 2011, 18.0% in 2012, and 19.3% in 2013. Two markers distributed on chromosomes 2 and 10 were associated with GT (Table 3), of which RM573_Chr 2 had the highest average PVE of 6.4% over 4 years. Three markers distributed on chromosomes 1 and 6 were associated with TGW (Table 3). More than 60% of the markers were located on chromosome 6, and the corresponding PVE ranged from 4.5% to 6.5%. RM345 had the maximum PVE, which was 5.9% in 2010, 5.1% in 2011, 6.5% in 2012 and 6.0% in 2013. Three markers were synchronously associated with two traits, with RM348 associated with GL and GW, RM345 associated with GL and TGW and RM528 associated with GW and TGW.

### Significant marker-trait association loci detected in each subpopulation

For GL, the number of significant marker-trait association loci detected in POP1, POP2, POP3, POP4, POP5 and POP6 were 2, 1, 7, 6, 8 and 5, respectively. Among them, the loci RM3766, RM335, RM348, RM276, RM345, RM6976, RM201, RM6544 and RM209 were simultaneously detected in two different subpopulations (Supplementary Table 6). All 9 of these loci were also detected across the entire population except for RM209.

For GW, a total of 22 significant marker-trait association loci were detected in the 6 subpopulations, and the values from POP1 to POP6 were 5, 1, 6, 4, 4 and 2. The loci RM348, RM528 and RM6863 were simultaneously detected in three different subpopulations, and the loci RM1019, RM528 and RM209 were simultaneously detected in two different subpopulations (Supplementary Table 6). Among the 6 aforementioned loci, RM348, RM528, RM1019 and RM6863 were also detected across the entire population.

For GT, only one locus was detected in each subpopulation. The locus RM573 was detected in four different subpopulations, and RM269 was detected in two different subpopulations (Supplementary Table 6). Both RM573 and RM269 were also detected across the entire population.

For TGW, the number of significant marker-trait association loci detected in POP1, POP2, POP3, POP4, POP5 and POP6 were 4, 1, 3, 1, 2 and 1, respectively. Among the 12 loci, RM345 was simultaneously detected in three different subpopulations, and RM1019, RM528, RM168 and RM490 were simultaneously detected in two different subpopulations (Supplementary Table 6). Only three loci—RM345, RM528 and RM490—were detected across the entire population.

### Discovery of elite alleles

In this study, the alleles with positive effects are considered elite alleles for all four grain traits measured. A summary of the elite alleles and their typical carrier materials is shown in Supplementary Table 7. The total numbers of elite alleles for GL, GW, GT and TGW detected across the entire population were 17, 10, 6 and 8, respectively. The allele RM335-155 bp showed the largest phenotypic effect (1.45 mm) for GL, and the typical carrier accession was Yuedao 32 (Supplementary Table 7). The allele RM348-145 bp showed the largest phenotypic effect (0.35 mm) for GW, and the typical carrier accession was Hongmangjing (Supplementary Table 7). The allele RM269-170 bp showed the largest phenotypic effect (0.14 mm) for GT, and the typical carrier accession was Wanhuangdao (Supplementary Table 7). The allele RM528-185 bp showed the largest phenotypic effect (0.67 g) for TGW, and the typical carrier accession was Xiangjing 9407 (Supplementary Table 7).

### Optimal cross designs for improving grain traits

**Figure 4:**
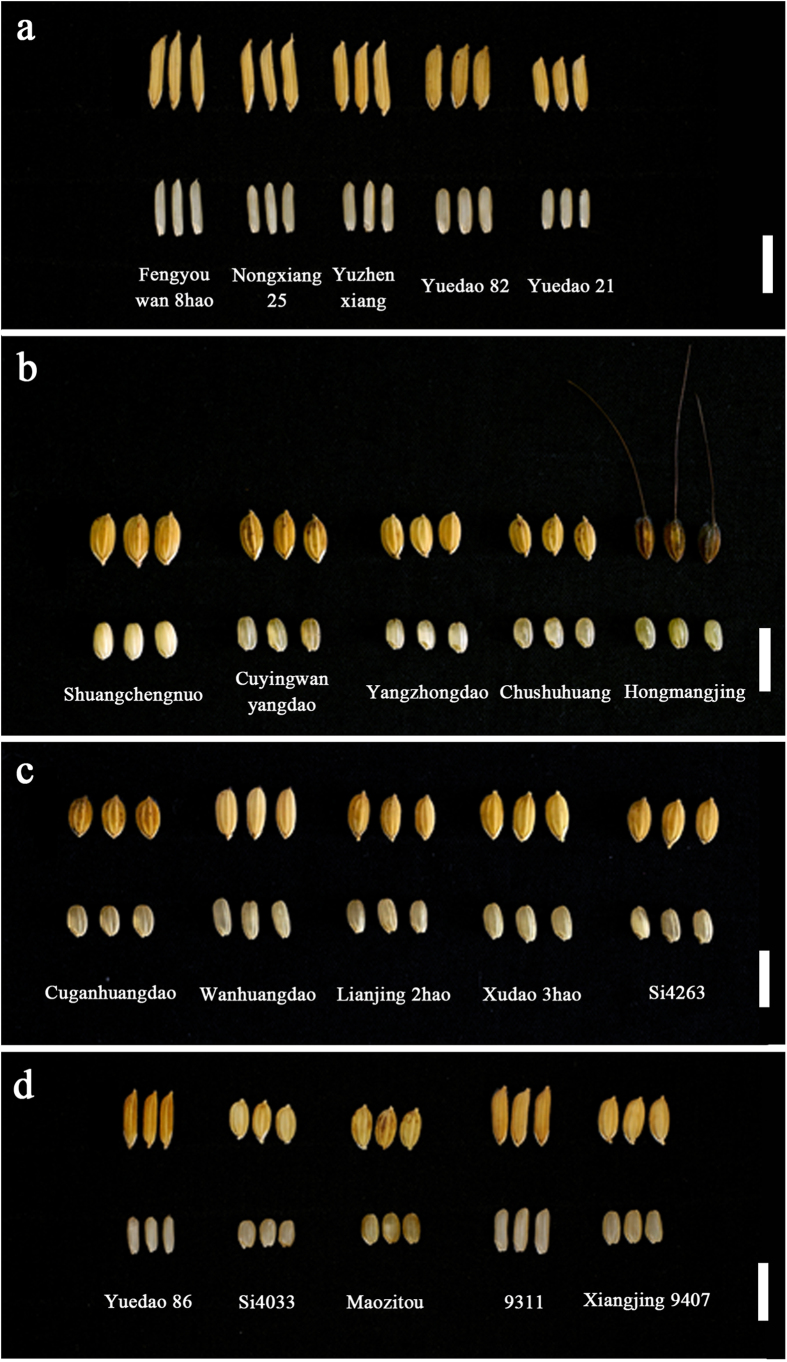
Unhulled grains (above) and brown rice (down) of the elite parents for improving of the 4 traits. **a** the five accessions for improving grain length (GL); **b** the five accessions for improving grain width (GW); **c** the five accessions for improving grain thickness (GT); **d** the five accessions for improving 1,000-grain weight (TGW).(bar = 10 mm).

Based on the number of elite alleles that could be substituted into an individual plant and the expected phenotypic effects of elite alleles that could be pyramided, the top five cross combinations for improving GL, GW, GT and TGW were proposed (Supplementary Table 8). The elite alleles carried by the parents in excellent crosses are listed in Supplementary Table 9. Figure 4 shows the five parents in the superior cross for each trait. Certain accessions were found repeatedly in the supposed parental combinations (e.g., Yuedao 21 and Cuyingwanyangdao emerged four times in the combinations for GL and GW), indicating that these accessions possess unique elite alleles.

## Discussion

The population genetic structure analysis showed that the six subpopulations occurred in the 532 accessions using the model-based method (Structure) and Nei’s genetic distance method. The results showed that POP1 consists of accessions collected from Southeast Asia (Vietnam, latitudes lower than 17°N) and belongs to the subspecies *indica*; and POP2 consists of accessions collected from East Asia (northeastern China and Japan, latitudes higher than 45°N) and belongs to the subspecies *japonica*. *Indica* rice is primarily grown in lowland regions throughout tropical Asia, whereas *japonica* is typically found in temperate East Asia, upland areas of Southeast Asia, and high altitude regions in South Asia. We found that the average standardized individual SSR allele in POP1 and POP6 consisted primarily of low-latitude accessions from Vietnam and southern China, respectively, and these alleles were significantly smaller than those of POP2 and POP4, the high-latitude accessions from northeastern China and northern China (mainly *Japonica* subspecies). This result suggests a tendency of directional evolution in *O. sativa* from low (subspecies *indica*) to high latitudes (subspecies *japonica*). No remarkable difference between POP6 (mainly modern *indica* cultivars from southern China) and POP1 could explain the short geographical distance between the two subpopulations. The average individual SSR allele in POP3 (consisting primarily of *japonica* landraces from the Taihu Lake valley) was significantly shorter than those in POP2 and POP4, and this may reflect the directional evolution of *O. sativa* from low latitudes (warmer climate) to high latitudes (cooler climate) if the independent domestication of *indica* and *japonica* were true^2^.

In the present study, we define a rice accession with local adaptation, long history of cultivation, tall stature, susceptible to pathogen and pest epidemic currently, lack of formal genetic improvement as a rice landraces, which is somewhat different from the definition of landrace proposed by Villa *et al.*^38^, since rice is a strictly self-pollinated crop, and genetic diversity of intra-accession do not exist.

The proportion of rare alleles (frequency less than 5%) was 36.25% within the 2698 identified alleles. The high ratio of rare alleles in the present study might have been caused by the wide distribution of latitudes of the accessions. As the rice cultivation area expands from south to north and from the plains to plateau, new alleles appeared and certain original alleles disappeared, resulting in the emergence of accessions with rare alleles.

The average number of alleles per locus was 10.46 among the 532 accessions genotyped with 258 markers. The allele number per locus is higher than that reported by Garris *et al.*^37^, Agrama *et al.*^39^, and Vanniaraja *et al.*^40^ but lower than that reported by Borba *et al.*^41^ and Li *et al.*^42^. The average PIC value in this study was 0.7042, which is the highest value among previous studies for rice populations^41^,^43^, with an exception of the PIC value of 0.71 reported by Li *et al.*^42^ and PIC value of 0.75 reported by Borba *et al.*^44^. The wide range of genetic diversity and manageable number of accessions in this study indicate that this is one of the best data sets for mining valuable genes in rice.

An association analysis that does not consider population structure would have a high rate of Type I errors (false positive). In this study, the 532 *O. sativa* accessions were classified into six model-based subpopulations based on an ancestry analysis (Fig. 1a). The dendrogram (Fig. 1b) based on Nei’s genetic distance was consistent with the population structure determined by this collection. Therefore, the results obtained from these two separate analyses are consistent. In addition, the population structure was dependent on geographic origin, such as the accessions from Vietnam, which were classified into POP1, and the accessions from northeastern China, most of which were classified into POP2. The distinct geographic origins that correspond to the different ecological environments could be partially responsible for the observed genetic differentiation, which in turn contributes to the different responses to environmental factors and rare alleles in the germplasm accessions.

The significant *F*_ST_ values among the subpopulations (Table 1) suggest a divergence between these subpopulations, and heterosis might be observed for the crosses between the accessions to improve yield.

The number of markers required to cover the genome in an association study is determined by the extent of LD. To date, varying results have been reported for LD patterns by researchers in rice. Olsen *et al.*^45^ and Mather *et al.*^46^ reported LD decay occurring at an approximately 1 cM distance using DNA sequencing, whereas Dang *et al.*^30^, Agrama *et al.*^39^, and Vanniarajan *et al.*^40^ reported LD decay at 10–80 cM, 20–30 cM and 20–30 cM distances, respectively, using SSR markers. Compared with previous studies, the LD values for POP1-POP6 in the present study were similar to those reported by Vanniarajan *et al.*^40^. The variation in LD patterns across chromosomal regions observed at the subpopulation level suggests that the extent of LD varies among different rice accessions^39^, different markers^45^ and different genomic regions^46^. In this study, POP1 presented the fastest decay velocity, and it was followed by PO5, POP2, POP3, POP4 and POP6. The fast observed decay might have been caused by frequent artificial hybridization that is used in breeding because of the short day length in the lower latitudes of the Northern Hemisphere. The materials in POP2, POP3, POP4 and POP6 were mostly superior and modern accessions, and the chance for improvement was limited; therefore, they presented a slow decay velocity.

In this study, we identified seventeen markers associated with grain traits using the entire set of accessions, including 8 associated with GL, 4 associated with GW, 2 associated with GT and 3 associated with TGW. Nine of the 17 associations were in regions where the QTL associated with the given trait had been identified ( http://www.gramene.org/), and they are listed in Supplementary Table 10. Eight loci in this study were found for the first time, including 4 for GL, 3 for GW and 1 for GT. For the 4 new loci in GL, RM335_Chr 4 had the largest PVE (12.9% in 2010, 12.8% in 2011, 13.1% in 2012 and 10.8% in 2013) (Table 3). For the 3 new loci in GW, the PVE averaged ranged from 2.4% to 16.1% over 4 years. The marker RM573, which is located on chromosome 2, was a new locus associated with GT, and it showed a PVE from 5.0% to 8.0% over 4 years. These results might increase the descriptive power of the QTLs associated with the grain traits in rice and provide useful information for further fine mapping or cloning. In addition, certain loci were mapped at close to gene resolution (e.g., RM6976 close to *GS3*), indicating that association analyses of rice accessions can provide an effective approach for gene identification.

After comparing the results of the significant marker-trait loci between the subpopulations and the entire population, two phenomena were observed. The first phenomenon is that the PVE for the same significant marker-trait loci detected in the subpopulations was larger than that detected across the entire population (Table 3, Supplementary Table 6). For instance, the PVE of the locus RM345 for GL detected across 4 years was 23.1%, 21.2%, 32.5% and 17.9% in POP4 and 30.2%, 31.1%, 31.0% and 21.5% in POP6, whereas the PVE of the locus RM345 for GL detected across the entire population for 4 years was 4.8%, 3.9%, 6.1% and 6.3% (Table 3, Supplementary Table 6). The second phenomenon is that the 17 significant marker-trait loci detected across the entire population were not detected in a single subpopulation but in different subpopulations. For example, the locus RM6863 for GW was detected in POP2, POP3 and POP5, but not in POP1, POP4 and POP6, and the locus RM269 for GT was detected in POP1, POP4, POP5 and POP6 but not in POP2 and POP3 (Table 3, Supplementary Table 6). These phenomena might be interpreted as the smoothing effect caused by population size between the entire population and subpopulations in the marker-trait association analysis. The smoothing effect means that the trait difference, which was significant within a subpopulation, may become insignificant when the subpopulation accessions are integrated into the entire population.

Comparing the results of the significant marker-trait loci among the six subpopulations, we found that certain loci were simultaneously detected in more than 2 subpopulations, but no single locus was simultaneously detected in all 6 subpopulations. This phenomenon might be interpreted as the difference in genetic differentiation among the subpopulations.

To maintain information integrity, the results of significant marker-trait association loci obtained from the entire population were used for mining elite alleles. Seventeen elite alleles for GL were mined at the eight identified loci. Among them, 17.6% of the elite alleles were carried by accessions collected from northeastern China, 29.4% were carried by accessions from central China, and 52.9% were carried by accessions from Vietnam. Similarly, certain unique elite alleles were identified in various accessions for GW, GT and TGW, and these results suggest that during the process of rice evolution from the south to north, certain alleles were lost in the process of natural or artificial selection, whereas others were retained in modern cultivars or appeared for the first time in modern cultivars. For example, RM345-165 bp was common for Vietnam accessions but not found in northeastern China accessions, whereas the allele RM345-150 bp was found only in northeastern China accessions.

Correlations between the measured traits were observed, and GL was significantly negatively correlated with GW but positively correlated with TGW. We identified one SSR marker co-associated with GL and TGW in which the allele RM345-150 bp increased the phenotypic effect values of GL and TGW simultaneously (Table 3, Supplementary Table 7). We also detected one SSR marker co-associated with GL and GW in which the alleles RM348-130 bp, RM348-145 bp and RM348-170 bp increased with GW but decreased with GL (Table 3, Supplementary Table 7). These co-associated alleles have the correct sign with respect to trait correlations, and these data illustrate the genetic basis of trait correlations. In addition, if grain length elongates rapidly, then it will likely consume more carbohydrates in the endosperm, thereby resulting in less supply for the grain width, and vice versa.

For the GL trait, the broad-sense heritability averaged across four years was 97%, which was considerably high. Thus, the expected results for improving GL could be obtained by marker-assisted selection. Among the eight SSR-associated markers detected for GL, RM335_Chr 4 had the largest PVE (12.9%, 12.8%, 13.1% and 10.8% in 2010–2013) and among the two elite alleles found at this marker locus, RM335-155 bp had the largest phenotypic effect value (1.45 mm). This elite allele was carried by 45 accessions, and Yuedao 32 was the typical carrier material. Thus, GL could be improved greatly by the crosses described in Supplementary Table 8.

For the GW trait, the broad-sense heritability averaged across four years was 95%. Among the four SSR-associated markers detected for GW, RM1019_Chr 8 had the largest PVE (15.2%, 12.1%, 8.0% and 9.0% in 2010–2013), and among the four elite alleles found at this marker locus, RM1019-150 bp had the largest phenotypic effect value (0.28 mm). This elite allele was carried by 64 accessions, and Hongmangjing was the typical carrier material. Thus, GW could be improved greatly by the crosses described in Supplementary Table 8.

For the GT trait, the broad-sense heritability averaged across four years was 93%. Among the two SSR-associated markers detected for GT, RM573_Chr 2 had the largest PVE (5.6%, 5.0%, 6.9% and 8.0% in 2010–2013), and among the four elite alleles found at this marker locus, RM573-220 bp had the largest phenotypic effect value (0.08 mm). This elite allele was carried by 32 accessions, and Si4263 was the typical carrier material. Thus, GT could be improved greatly by the crosses described in Supplementary Table 8.

The broad-sense heritability averaged across four years for the TGW trait was 94%, which was also high. Among the three SSR markers associated with TGW, RM345_Chr 6 had the largest PVE (5.9%, 5.1%, 6.5% and 6.0% in 2010-2013, respectively), and four elite alleles—RM345-105 bp, RM345-150 bp, RM573-155 bp and RM345-160 bp—were found at this marker locus. Thus, TGW might be improved by the crosses listed in Supplementary Table 8.

If the target trait must be further improved, the best elite alleles could be pyramided into one cultivar using multi-round crossing. For example, there were 17 elite alleles detected for GL, and the 8 best elite alleles could be pyramided or substituted by the combination of the accessions Yuzhenxiang, Yuedao 82, Yuedao 21, Fengyouwan 8hao, and Nongxiang 25 (Supplementary Table 8).

## Materials and methods

### Plant materials

A total of 532 rice accessions from the geographical regions of East and Southeast Asia were used for the association mapping, including 121 from Vietnam (17°N – 23°N), 400 from China and 11 from Japan (20°N – 54°N). Detailed information on their origins is summarized in Supplementary Table 2.

### Field planting and trait measurement

The 532 accessions were planted in a paddy rice field at the Nanjing Agricultural University Experimental Farm, Nanjing, China from May to October in 2010, 2011, 2012 and 2013. The field experiments in the four consecutive years were treated as four independent environments. The field trials followed a completely randomized block design with two replicates per year. Each plot contained five rows, with 8 plants in each row, 17 cm between plants within each row and 20 cm between rows. The field management followed standard agricultural practices. At the mature stage, 5 normally developed plants from the middle of the plots were harvested each year and dried under natural conditions for the trait investigation. Fully filled grains were used for measuring the grain length (GL/mm), grain width (GW/mm), grain thickness (GT/mm) and 1,000-grain weight (TGW/g). Ten randomly chosen grains (after removing awns) from each plant were lined up length-wise along an electronic digital Vernier calliper to measure the grain length and then arranged by breadth to measure the grain width. The individual grain thickness was determined according to the maximal values for each grain using a Vernier calliper, and the values were averaged and used as the measurements for the plants. The 1,000-grain weight was calculated based on 1,000 grains.

### SSR marker genotyping

Genomic DNA was extracted from the leaf tissue of one single plant in each plot (the plants within a plot were homogeneity) according to the methods described by Murray and Thompson^47^. According to the published rice molecular map and microsatellite database of Temnykh *et al.*^48^ and McCouch *et al.*^49^, 258 SSRs scattered on 12 chromosomes were selected. The primers were synthesized by Shanghai Generay Biotech Co. Ltd., Shanghai, China. Each 10 μl PCR reaction contained 10 mM Tris-HCl (pH 9.0), 50 mM KCl, 0.1% Triton X-100, 1.5 mM MgCl_2_, 0.5 nM dNTPs, 0.14 pM forward primers, 0.14 pM reverse primers, 0.5 units Taq polymerase, and 20 ng genomic DNA. The DNA amplification was performed using a PTC-100^TM^ Peltier Thermal Cycler (MJ Research^TM^ Incorporated, USA) under the following conditions: 1) denaturation at 94 °C for 5 min; 2) 34 cycles of denaturation at 94 °C for 0.5 min, annealing at 55–63 °C for 1 min, and extension at 72 °C for 1 min; and 3) final extension at 72 °C for 10 min. The PCR products were run on an 8% polyacrylamide gel at 150 V for 1 h and visualized using silver staining. One pair of SSR markers detected one locus, and each polymorphic band at the same marker locus in the population was recorded as one allele. After screening the polyacrylamide gel electrophoresis (PAGE) products, the molecular weight of each band was calculated by the software Quantity One.

### Data analysis

All of the basic statistical analyses were performed using the SAS package (SAS Institute Inc., Cary, NC, USA). Broad-sense heritability (H^2^_B_) was calculated according to the natural population through an analysis of variance using the formula H^2^_B_ = σ^2^_g_/ (σ^2^_g_ + σ^2^_e_/n), where σ^2^_g_ is the genetic variance, σ^2^_e_ is the error variance, and n is the number of replicates.

The PIC value was used to measure the allele diversity at a locus, and the allele number per locus and genetic diversity were calculated using PowerMarker version 3.25 software^50^ to quantify the genetic variation within the 532 accessions. Nei’s^51^ distance was also calculated and used for the unrooted phylogeny reconstruction using the neighbour-joining method as implemented in PowerMarker with the tree viewed using MEGA 4.0^52^.

Levels of genetic variation within and among populations identified by the subpopulation analysis were estimated from allelic frequencies using AMOVA^53^. The software Arlequin 3.01^54^ was used to perform the AMOVA procedure using SSR and standard multi-locus frequency data.

The software SPAGeDi^55^ (Spatial Pattern Analysis of Genetic Diversity) was used to calculate the pairwise relatedness coefficients (K, kinship matrix) to estimate the genetic relatedness among individuals, and the negative value of kinship set to zero.

We calculated the average individual allele size of the SSRs as the mean of the standardization size of the 258 SSR loci following the method reported by Vigouroux *et al.*^56^.

### Population structure analysis

The optimum number of populations (K) was selected after five independent runs of a burn-in of 50,000 iterations followed by 100,000 iterations for each value of K (from 2 to 10) using Structure version 2.2^57^. The mean log-likelihood value over 5 runs at each K value was used. If the mean log-likelihood value reached the highest value in the model parameter K, a suitable value of K was determined. The non-admixed individuals (accession that could be clearly assigned to only one group) in each genetic subpopulation were determined using the Q-matrix assignment of above 0.9.

### Linkage disequilibrium

LD was estimated by the D' value^58^ between all pairs of SSRs with 1,000 permutations and calculated using TASSEL 3.0 software^59^. Rare alleles with an allele frequency of 5% or less were removed from the dataset before the association analysis. According to the level of LD and genetic distance among markers with intrachromosomal combinations, the regression equation of LD with the genetic distance changes was calculated by the regression analysis. The LD decay plot was drawn to observe the relation between LD and genetic distance.

### Association mapping

The associations between traits and markers were calculated using an MLM as described in TASSEL 3.0^59^. An MLM can significantly reduce spurious marker-trait associations (Type I error showing false positives) resulting from the population structure because Q and K matrices are used as covariants in the analysis. The Q matrix was adapted from the analysis results obtained from Structure 2.2. The K matrix (kinship matrix) was obtained from the result of the relatedness analysis using SPAGeDi. A false discovery rate (FDR) of 0.05 was used as a threshold for significant associations using the Benjamini and Hochberg^60^ correction method. Using the identified association locus, the ‘null allele’ (non-amplified allele) was used to determine the phenotypic effects of other alleles^32^.

## Additional Information

**How to cite this article**: Dang, X. *et al.* Population genetic structure of *Oryza sativa* in East and Southeast Asia and the discovery of elite alleles for grain traits. *Sci. Rep.* **5**, 11254; doi: 10.1038/srep11254 (2015).

## Supplementary Material

Supplementary Information

## Figures and Tables

**Table 1:** Pairwise estimates of *F*_ST_ based on 258 SSR loci among the six subpopulations.

**Cluster**	**POP1**	**POP2**	**POP3**	**POP4**	**POP5**	**POP6**
POP1	–					
POP2	0.456	–				
POP3	0.353	0.284	–			
POP4	0.366	0.196	0.142	–		
POP5	0.435	0.305	0.248	0.226	–	
POP6	0.380	0.263	0.221	0.155	0.256	–

**Table 2:** Phenotypic characteristics for grain traits in 532 rice accessions across 4 years.

**Traits**	**Year**	**Maximum**	**Minimum**	**Mean**	**Standard deviation**	**Skewness**	**Kurtosis**	**Heritability in the broad sense %**
Grain length (mm)	2010	12.91	6.44	8.09	0.99	1.68	3.60	98.76
	2011	12.71	6.26	8.10	0.97	1.62	3.54	98.88
	2012	13.27	6.40	8.11	1.02	1.64	3.45	99.07
	2013	12.78	6.42	7.97	1.08	1.71	3.34	96.33
Grain width (mm)	2010	4.39	2.22	3.21	0.40	−0.67	−0.08	96.17
	2011	4.38	2.06	3.20	0.42	−0.62	−0.14	95.79
	2012	4.42	2.21	3.18	0.37	−0.51	−0.20	97.87
	2013	4.04	1.98	3.14	0.43	−0.75	−0.40	93.60
Grain thickness (mm)	2010	2.94	1.79	2.22	0.18	0.36	0.96	97.37
	2011	3.00	1.68	2.21	0.19	0.12	0.93	96.88
	2012	3.12	1.71	2.21	0.20	0.45	0.56	95.65
	2013	3.07	1.73	2.20	0.20	0.42	0.39	95.34
1,000-grain weight (g)	2010	33.23	17.03	24.79	2.58	−0.11	0.15	96.19
	2011	32.29	16.21	24.95	2.52	−0.09	0.04	97.44
	2012	32.75	15.73	24.89	2.49	−0.09	0.23	95.97
	2013	32.62	16.17	24.59	2.54	−0.05	0.14	92.52

**Table 3:** Marker-trait associations with *P*-value less than 0.05, their equivalent false discovery rate probability (FDR), proportion of phenotypic variance explained (PVE), marker position on chromosome derived from 258 markers and 474 rice accessions.

**Traits**	**Markers**	**Chr.**	**Position /cM**	**2010**			**2011**			**2012**			**2013**		
				***P*** **value**	**PVE**	**FDR**	***P*** **value**	**PVE**	**FDR**	***P*** **value**	**PVE**	**FDR**	***P*** **Value**	**PVE**	**FDR**
GL	RM3766	3	34.8	6.98E-04	0.063	1.17E-02	5.57E-04	0.064	1.17E-02	1.60E-03	0.050	1.67E-02	4.93E-03	0.051	1.67E-02
	RM335	4	5.4	8.49E-09	0.129	1.67E-03	1.01E-08	0.128	1.67E-03	5.66E-09	0.131	1.85E-03	4.02E-07	0.108	3.03E-03
	RM348	4	160.8	3.15E-03	0.025	1.67E-02	4.02E-03	0.024	1.83E-02	2.93E-03	0.025	1.85E-02	2.64E-02	0.016	4.09E-02
	RM276	6	33.5	7.33E-04	0.067	1.33E-02	2.16E-03	0.061	1.33E-02	1.15E-03	0.064	1.48E-02	2.17E-02	0.045	3.79E-02
	RM345	6	123.9	4.39E-04	0.048	1.00E-02	2.58E-03	0.039	1.50E-02	3.26E-05	0.061	7.41E-03	2.37E-05	0.063	6.06E-03
	RM6976	8	92.2	1.06E-04	0.100	6.67E-03	1.99E-04	0.095	6.67E-03	2.59E-05	0.109	5.56E-03	2.72E-07	0.117	1.52E-03
	RM201	9	81.2	3.54E-04	0.060	8.33E-03	2.68E-04	0.061	8.33E-03	4.44E-04	0.059	1.30E-02	4.02E-03	0.046	1.52E-02
	RM6544	11	19.8	6.28E-05	0.057	5.00E-03	7.81E-05	0.056	5.00E-03	1.48E-05	0.066	3.70E-03	1.24E-06	0.075	4.55E-03
GW	RM348	4	160.8	7.16E-04	0.031	4.84E-03	6.28E-03	0.022	1.54E-02	9.01E-03	0.021	1.35E-02	7.77E-03	0.021	2.32E-02
	RM528	6	100.8	2.02E-02	0.065	3.06E-02	8.88E-03	0.052	2.12E-02	1.18E-03	0.087	7.69E-03	2.42E-03	0.074	1.25E-02
	RM1019	8	0.5	2.02E-07	0.152	1.61E-03	1.44E-05	0.121	1.92E-03	1.84E-03	0.180	9.62E-03	1.60E-03	0.193	1.07E-02
	RM6863	8	16.4	8.32E-03	0.042	2.10E-02	9.15E-04	0.055	7.69E-03	3.36E-02	0.053	3.85E-02	6.64E-08	0.048	1.79E-03
GT	RM573	2	118.1	1.10E-02	0.056	1.25E-02	1.99E-02	0.050	2.19E-02	1.71E-03	0.069	5.26E-03	1.72E-04	0.080	2.63E-03
	RM269	10	69.6	9.62E-04	0.036	6.25E-03	8.55E-04	0.036	6.25E-03	1.36E-03	0.034	2.63E-03	5.88E-03	0.028	1.32E-02
TGW	RM490	1	51	1.70E-02	0.045	1.82E-02	1.45E-02	0.046	2.14E-02	9.97E-03	0.049	1.67E-02	5.98E-03	0.062	1.25E-02
	RM528	6	100.8	4.05E-02	0.058	4.55E-02	3.16E-02	0.050	3.21E-02	3.82E-02	0.052	4.17E-02	8.93E-03	0.048	3.75E-02
	RM345	6	123.9	5.51E-05	0.059	4.55E-03	2.58E-04	0.051	3.57E-03	1.49E-05	0.065	4.17E-03	3.88E-03	0.060	6.25E-03
