# Supplementary material for: Population genetic structure of Oryza sativa in East and Southeast Asia and the discovery of elite alleles for grain traits
Source: Sci Rep. 2015 Jun 10;5:11254. doi: 10.1038/srep11254 (PMC4462027; doi:10.1038/srep11254)
Supplement: Supplementary Information [file srep11254-s1.doc]

**Population genetic structure of *Oryza sativa* in East and Southeast Asia and the discovery of elite allele for grain traits**

**Xiaojing Dang, Thu Giang Tran Thi, Wisdom Mawuli Edzesi, Lijun Liang, Qiangming Liu, Erbao Liu, Yang Wang, Sheng Qiang, Linglong Liu, Delin Hong***

Supplementary Tables 1-10

Supplementary Figures 1-2

**Supplementary Table 1.** Summary statistics for the 258 SSR markers used in this study.

| Code | Locus | Chr. No. | Position/cM | Allele number | Gene diversity | PIC |  | Code | Locus | Chr. No. | Position/cM | Allele number | Gene diversity | PIC |
| --- | --- | --- | --- | --- | --- | --- | --- | --- | --- | --- | --- | --- | --- | --- |
| 1 | RM84 | 1 | 18.8 | 7 | 0.4364 | 0.4086 |  | 131 | RM136 | 6 | 53.0 | 19 | 0.8672 | 0.8552 |
| 2 | RM1-003 | 1 | 19.9 | 12 | 0.8269 | 0.8064 |  | 132 | RM3330 | 6 | 61.6 | 14 | 0.8840 | 0.8740 |
| 3 | RM283 | 1 | 25.4 | 8 | 0.8433 | 0.8234 |  | 133 | RM3187 | 6 | 73.2 | 6 | 0.6970 | 0.6438 |
| 4 | RM3453 | 1 | 25.4 | 14 | 0.8620 | 0.8489 |  | 134 | RM7579 | 6 | 84.5 | 6 | 0.7420 | 0.6949 |
| 5 | RM1 | 1 | 29.7 | 11 | 0.7894 | 0.7676 |  | 135 | RM8239 | 6 | 91.9 | 5 | 0.7352 | 0.6905 |
| 6 | RM259 | 1 | 38.8 | 21 | 0.8809 | 0.8705 |  | 136 | RM454 | 6 | 99.3 | 4 | 0.5270 | 0.4720 |
| 7 | RM583 | 1 | 43.2 | 6 | 0.7045 | 0.6501 |  | 137 | RM7309 | 6 | 100.3 | 16 | 0.8564 | 0.8434 |
| 8 | RM490 | 1 | 51.0 | 5 | 0.2995 | 0.2834 |  | 138 | RM528 | 6 | 100.8 | 11 | 0.7456 | 0.7107 |
| 9 | RM8095 | 1 | 60.6 | 9 | 0.8301 | 0.8082 |  | 139 | RM3138 | 6 | 110.6 | 12 | 0.8385 | 0.8198 |
| 10 | RM140 | 1 | 65.4 | 8 | 0.2892 | 0.2780 |  | 140 | RM162 | 6 | 114.9 | 21 | 0.9077 | 0.9008 |
| 11 | RM562 | 1 | 78.4 | 21 | 0.9208 | 0.9152 |  | 141 | RM6811 | 6 | 115.6 | 15 | 0.9009 | 0.8925 |
| 12 | RM9 | 1 | 92.4 | 16 | 0.8188 | 0.7988 |  | 142 | RM345 | 6 | 123.9 | 6 | 0.5957 | 0.5374 |
| 13 | RM129 | 1 | 93.0 | 5 | 0.3227 | 0.3110 |  | 143 | RM5753 | 6 | 124.4 | 21 | 0.8729 | 0.8637 |
| 14 | RM5 | 1 | 98.5 | 10 | 0.8332 | 0.8115 |  | 144 | RM295 | 7 | 0.0 | 12 | 0.6904 | 0.6627 |
| 15 | RM1231 | 1 | 123.2 | 16 | 0.9016 | 0.8936 |  | 145 | RM125 | 7 | 24.8 | 4 | 0.5615 | 0.4646 |
| 16 | RM128 | 1 | 126.5 | 16 | 0.8445 | 0.8291 |  | 146 | RM180 | 7 | 30.1 | 8 | 0.6279 | 0.5917 |
| 17 | RM246 | 1 | 134.6 | 16 | 0.8682 | 0.8543 |  | 147 | RM542 | 7 | 34.7 | 7 | 0.8011 | 0.7719 |
| 18 | RM212 | 1 | 135.8 | 5 | 0.4144 | 0.3913 |  | 148 | RM8263 | 7 | 35.7 | 8 | 0.6647 | 0.6101 |
| 19 | RM5389 | 1 | 142.4 | 13 | 0.8602 | 0.8446 |  | 149 | RM418 | 7 | 42.1 | 9 | 0.7251 | 0.6837 |
| 20 | RM486 | 1 | 153.5 | 10 | 0.7205 | 0.6780 |  | 150 | RM346 | 7 | 47.0 | 9 | 0.7763 | 0.7480 |
| 21 | RM265 | 1 | 155.9 | 8 | 0.7236 | 0.6875 |  | 151 | RM2530 | 7 | 53.4 | 15 | 0.8759 | 0.8643 |
| 22 | RM3482 | 1 | 157.6 | 14 | 0.8432 | 0.8303 |  | 152 | RM336 | 7 | 61.0 | 12 | 0.8200 | 0.8007 |
| 23 | RM6831 | 1 | 181.8 | 9 | 0.7538 | 0.7182 |  | 153 | RM5380 | 7 | 67.0 | 10 | 0.7719 | 0.7359 |
| 24 | RM14 | 1 | 194.0 | 14 | 0.8775 | 0.8662 |  | 154 | RM6011 | 7 | 73.2 | 14 | 0.8952 | 0.8861 |
| 25 | RM5340 | 2 | 36.3 | 16 | 0.8776 | 0.8661 |  | 155 | RM505 | 7 | 78.6 | 9 | 0.8306 | 0.8093 |
| 26 | RM7288 | 2 | 42.4 | 23 | 0.9033 | 0.8959 |  | 156 | RM3589 | 7 | 89.8 | 13 | 0.8188 | 0.7973 |
| 27 | RM5356 | 2 | 43.3 | 10 | 0.8470 | 0.8283 |  | 157 | RM11 | 7 | 93.8 | 8 | 0.7659 | 0.7319 |
| 28 | RM1358 | 2 | 48.1 | 10 | 0.8244 | 0.8011 |  | 158 | RM234 | 7 | 93.9 | 14 | 0.8168 | 0.7994 |
| 29 | RM1313 | 2 | 51.1 | 10 | 0.8037 | 0.7764 |  | 159 | RM134 | 7 | 99.6 | 10 | 0.8370 | 0.8174 |
| 30 | RM324 | 2 | 51.1 | 5 | 0.4250 | 0.3845 |  | 160 | RM1306 | 7 | 116.1 | 13 | 0.8400 | 0.8261 |
| 31 | RM327 | 2 | 51.9 | 11 | 0.8411 | 0.8215 |  | 161 | RM82 | 7 | 128.9 | 4 | 0.2761 | 0.2619 |
| 32 | RM301 | 2 | 53.5 | 8 | 0.7832 | 0.7536 |  | 162 | RM506 | 8 | 0.0 | 12 | 0.7953 | 0.7723 |
| 33 | RM300 | 2 | 54.6 | 15 | 0.8821 | 0.8709 |  | 163 | RM1019 | 8 | 0.5 | 18 | 0.8740 | 0.8625 |
| 34 | RM262 | 2 | 70.2 | 10 | 0.7770 | 0.7455 |  | 164 | RM152 | 8 | 9.4 | 13 | 0.8124 | 0.7894 |
| 35 | RM5427 | 2 | 84.6 | 12 | 0.6988 | 0.6545 |  | 165 | RM1235 | 8 | 12.8 | 4 | 0.5962 | 0.5242 |
| 36 | RM3688 | 2 | 88.2 | 8 | 0.8524 | 0.8347 |  | 166 | RM6863 | 8 | 16.4 | 11 | 0.7900 | 0.7597 |
| 37 | RM183 | 2 | 93.5 | 15 | 0.8616 | 0.8467 |  | 167 | RM4085 | 8 | 35.7 | 12 | 0.8553 | 0.8406 |
| 38 | RM5804 | 2 | 98.2 | 8 | 0.7773 | 0.7443 |  | 168 | RM544 | 8 | 38.5 | 15 | 0.9133 | 0.9067 |
| 39 | RM106 | 2 | 101.5 | 18 | 0.9018 | 0.8939 |  | 169 | RM8243 | 8 | 50.8 | 12 | 0.8792 | 0.8669 |
| 40 | RM6361 | 2 | 102.9 | 8 | 0.7476 | 0.7129 |  | 170 | RM25 | 8 | 52.2 | 12 | 0.7790 | 0.7504 |
| 41 | RM573 | 2 | 118.1 | 15 | 0.8368 | 0.8189 |  | 171 | RM331 | 8 | 59.0 | 11 | 0.8008 | 0.7728 |
| 42 | RM450 | 2 | 122.8 | 7 | 0.8104 | 0.7859 |  | 172 | RM72 | 8 | 60.9 | 14 | 0.8034 | 0.7846 |
| 43 | RM7598 | 2 | 126.4 | 7 | 0.5762 | 0.5525 |  | 173 | RM6215 | 8 | 66.8 | 11 | 0.7915 | 0.7691 |
| 44 | RM263 | 2 | 127.5 | 14 | 0.8636 | 0.8499 |  | 174 | RM7556 | 8 | 86.7 | 12 | 0.8438 | 0.8244 |
| 45 | RM112 | 2 | 137.5 | 9 | 0.6487 | 0.6213 |  | 175 | RM6976 | 8 | 92.2 | 19 | 0.8925 | 0.8840 |
| 46 | RM525 | 2 | 143.7 | 19 | 0.8541 | 0.8421 |  | 176 | RM80 | 8 | 103.7 | 13 | 0.6313 | 0.6135 |
| 47 | RM213 | 2 | 150.5 | 11 | 0.8409 | 0.8223 |  | 177 | RM502 | 8 | 109.3 | 15 | 0.8610 | 0.8464 |
| 48 | RM208 | 2 | 154.1 | 6 | 0.6481 | 0.5892 |  | 178 | RM3754 | 8 | 112.6 | 7 | 0.8145 | 0.7902 |
| 49 | RM3850 | 2 | 156.3 | 14 | 0.8501 | 0.8344 |  | 179 | RM6948 | 8 | 114.4 | 7 | 0.7238 | 0.6897 |
| 50 | RM498 | 2 | 156.3 | 19 | 0.8202 | 0.7991 |  | 180 | RM433 | 8 | 116.0 | 7 | 0.2576 | 0.2499 |
| 51 | RM48 | 2 | 191.2 | 10 | 0.4230 | 0.4096 |  | 181 | RM281 | 8 | 128.1 | 14 | 0.8361 | 0.8168 |
| 52 | RM266 | 2 | 192.2 | 11 | 0.7582 | 0.7288 |  | 182 | RM264 | 8 | 138.2 | 12 | 0.8633 | 0.8484 |
| 53 | RM535 | 2 | 195.7 | 17 | 0.8675 | 0.8557 |  | 183 | RM1328 | 9 | 0.0 | 14 | 0.8781 | 0.8659 |
| 54 | RM132 | 3 | 3.9 | 6 | 0.4027 | 0.3734 |  | 184 | RM8206 | 9 | 3.2 | 11 | 0.7405 | 0.7090 |
| 55 | RM1332 | 3 | 11.5 | 4 | 0.6879 | 0.6257 |  | 185 | RM524 | 9 | 42.5 | 12 | 0.8344 | 0.8142 |
| 56 | RM5849 | 3 | 18.4 | 15 | 0.8127 | 0.7903 |  | 186 | RM3912 | 9 | 46.3 | 10 | 0.8457 | 0.8261 |
| 57 | RM489 | 3 | 20.3 | 13 | 0.8926 | 0.8827 |  | 187 | RM566 | 9 | 50.7 | 9 | 0.7514 | 0.7135 |
| 58 | RM545 | 3 | 24.7 | 5 | 0.6122 | 0.5432 |  | 188 | RM434 | 9 | 57.7 | 10 | 0.8558 | 0.8400 |
| 59 | RM5480 | 3 | 25.9 | 11 | 0.6299 | 0.5984 |  | 189 | RM3600 | 9 | 62.7 | 12 | 0.7978 | 0.7732 |
| 60 | RM3467 | 3 | 28.2 | 14 | 0.8921 | 0.8825 |  | 190 | RM24481 | 9 | 63.0 | 19 | 0.8973 | 0.8891 |
| 61 | RM3766 | 3 | 34.8 | 10 | 0.7124 | 0.6820 |  | 191 | RM3533 | 9 | 65.1 | 13 | 0.8266 | 0.8053 |
| 62 | RM7 | 3 | 36.9 | 6 | 0.7162 | 0.6667 |  | 192 | RM6570 | 9 | 68.2 | 9 | 0.5426 | 0.5167 |
| 63 | RM5639 | 3 | 39.8 | 8 | 0.7273 | 0.6847 |  | 193 | RM410 | 9 | 79.3 | 9 | 0.7010 | 0.6578 |
| 64 | RM7197 | 3 | 44.4 | 11 | 0.8350 | 0.8143 |  | 194 | RM257 | 9 | 79.7 | 11 | 0.7715 | 0.7476 |
| 65 | RM7345 | 3 | 48.8 | 7 | 0.6728 | 0.6391 |  | 195 | RM201 | 9 | 81.2 | 9 | 0.7703 | 0.7362 |
| 66 | RM282 | 3 | 55.8 | 11 | 0.8483 | 0.8305 |  | 196 | OSR28 | 9 | 85.4 | 15 | 0.8584 | 0.8433 |
| 67 | RM338 | 3 | 61.9 | 8 | 0.7449 | 0.7066 |  | 197 | RM5384 | 9 | 90.7 | 10 | 0.8613 | 0.8458 |
| 68 | RM218 | 3 | 67.8 | 9 | 0.7140 | 0.6842 |  | 198 | RM1013 | 9 | 93.5 | 8 | 0.7635 | 0.7284 |
| 69 | RM232 | 3 | 76.7 | 13 | 0.8390 | 0.8213 |  | 199 | RM7492 | 10 | 0.0 | 12 | 0.8671 | 0.8536 |
| 70 | RM7403 | 3 | 82.3 | 4 | 0.2080 | 0.1904 |  | 200 | RM7545 | 10 | 7.6 | 25 | 0.9424 | 0.9394 |
| 71 | RM6266 | 3 | 94.9 | 6 | 0.6512 | 0.6078 |  | 201 | RM6646 | 10 | 13.3 | 9 | 0.8170 | 0.7971 |
| 72 | RM7097 | 3 | 115.6 | 7 | 0.7874 | 0.7556 |  | 202 | RM244 | 10 | 15.0 | 6 | 0.4846 | 0.4513 |
| 73 | RM135 | 3 | 120.4 | 9 | 0.7727 | 0.7422 |  | 203 | RM216 | 10 | 24.8 | 3 | 0.5998 | 0.5273 |
| 74 | RM168 | 3 | 122.8 | 12 | 0.5860 | 0.5598 |  | 204 | RM311 | 10 | 25.2 | 8 | 0.7980 | 0.7710 |
| 75 | RM186 | 3 | 127.4 | 7 | 0.7773 | 0.7408 |  | 205 | RM184 | 10 | 41.6 | 5 | 0.7560 | 0.7149 |
| 76 | RM16 | 3 | 131.5 | 7 | 0.3460 | 0.3149 |  | 206 | RM1125 | 10 | 46.8 | 14 | 0.8474 | 0.8332 |
| 77 | RM5475 | 3 | 137.9 | 21 | 0.9088 | 0.9018 |  | 207 | RM258 | 10 | 48.8 | 8 | 0.6829 | 0.6170 |
| 78 | RM416 | 3 | 140.1 | 9 | 0.7325 | 0.6948 |  | 208 | RM5629 | 10 | 53.6 | 11 | 0.7824 | 0.7554 |
| 79 | RM6712 | 3 | 158.2 | 11 | 0.7848 | 0.7599 |  | 209 | RM6100 | 10 | 53.9 | 5 | 0.3090 | 0.2863 |
| 80 | RM448 | 3 | 191.6 | 13 | 0.8725 | 0.8599 |  | 210 | RM1108 | 10 | 55.3 | 6 | 0.3176 | 0.3025 |
| 81 | RM148 | 3 | 249.3 | 8 | 0.7379 | 0.6921 |  | 211 | RM3773 | 10 | 58.9 | 17 | 0.8991 | 0.8905 |
| 82 | RM307 | 4 | 0.0 | 12 | 0.8475 | 0.8299 |  | 212 | RM269 | 10 | 69.6 | 5 | 0.6579 | 0.5946 |
| 83 | RM335 | 4 | 5.4 | 16 | 0.8406 | 0.8221 |  | 213 | RM5352 | 10 | 71.4 | 4 | 0.5312 | 0.4330 |
| 84 | RM518 | 4 | 7.9 | 9 | 0.7932 | 0.7641 |  | 214 | RM304 | 10 | 73.0 | 6 | 0.1423 | 0.1403 |
| 85 | RM3471 | 4 | 16.7 | 19 | 0.8902 | 0.8817 |  | 215 | RM171 | 10 | 73.0 | 5 | 0.6427 | 0.5740 |
| 86 | RM4835 | 4 | 18.3 | 9 | 0.5333 | 0.4478 |  | 216 | RM6160 | 10 | 81.0 | 8 | 0.6025 | 0.5654 |
| 87 | RM5687 | 4 | 25.4 | 9 | 0.7641 | 0.7376 |  | 217 | RM590 | 10 | 83.3 | 10 | 0.8134 | 0.7879 |
| 88 | RM6314 | 4 | 41.5 | 16 | 0.8288 | 0.8098 |  | 218 | RM333 | 10 | 110.4 | 7 | 0.3613 | 0.3490 |
| 89 | RM471 | 4 | 53.8 | 10 | 0.7842 | 0.7615 |  | 219 | RM286 | 11 | 0.1 | 8 | 0.7013 | 0.6706 |
| 90 | RM5951 | 4 | 56.1 | 5 | 0.7035 | 0.6518 |  | 220 | RM6327 | 11 | 1.7 | 20 | 0.8913 | 0.8815 |
| 91 | RM142 | 4 | 60.2 | 16 | 0.8717 | 0.8595 |  | 221 | RM1240 | 11 | 6.5 | 16 | 0.8599 | 0.8458 |
| 92 | RM6997 | 4 | 62.1 | 12 | 0.7680 | 0.7422 |  | 222 | RM7557 | 11 | 9.2 | 5 | 0.5824 | 0.5320 |
| 93 | RM7563 | 4 | 68.3 | 8 | 0.8496 | 0.8322 |  | 223 | RM1812 | 11 | 10.3 | 10 | 0.6100 | 0.5503 |
| 94 | RM6114 | 4 | 72.0 | 12 | 0.8606 | 0.8460 |  | 224 | RM6544 | 11 | 19.8 | 5 | 0.4867 | 0.4500 |
| 95 | RM6589 | 4 | 85.2 | 8 | 0.8364 | 0.8159 |  | 225 | RM3133 | 11 | 32.7 | 5 | 0.6854 | 0.6294 |
| 96 | RM317 | 4 | 96.0 | 6 | 0.7111 | 0.6611 |  | 226 | RM167 | 11 | 37.5 | 5 | 0.6594 | 0.5888 |
| 97 | RM6089 | 4 | 97.7 | 12 | 0.8389 | 0.8203 |  | 227 | RM3701 | 11 | 45.3 | 11 | 0.7264 | 0.6887 |
| 98 | RM3513 | 4 | 99.6 | 8 | 0.5337 | 0.4944 |  | 228 | RM7391 | 11 | 54.3 | 10 | 0.8260 | 0.8078 |
| 99 | RM3836 | 4 | 108.2 | 11 | 0.8438 | 0.8263 |  | 229 | RM7303 | 11 | 64.2 | 4 | 0.0775 | 0.0763 |
| 100 | RM280 | 4 | 128.9 | 3 | 0.3869 | 0.3357 |  | 230 | RM287 | 11 | 66.6 | 8 | 0.7654 | 0.7342 |
| 101 | RM559 | 4 | 129.6 | 6 | 0.7225 | 0.6793 |  | 231 | RM457 | 11 | 68.6 | 8 | 0.5801 | 0.5340 |
| 102 | RM349 | 4 | 146.8 | 8 | 0.7265 | 0.6914 |  | 232 | RM5349 | 11 | 78.8 | 6 | 0.6761 | 0.6270 |
| 103 | RM348 | 4 | 160.8 | 4 | 0.5227 | 0.4642 |  | 233 | RM209 | 11 | 79.1 | 6 | 0.4303 | 0.3869 |
| 104 | RM1182 | 5 | 3.0 | 11 | 0.8550 | 0.8382 |  | 234 | RM21 | 11 | 84.7 | 11 | 0.7617 | 0.7257 |
| 105 | RM153 | 5 | 3.0 | 12 | 0.8362 | 0.8183 |  | 235 | RM7170 | 11 | 85.7 | 12 | 0.8761 | 0.8635 |
| 106 | RM122 | 5 | 3.0 | 6 | 0.2219 | 0.2103 |  | 236 | RM206 | 11 | 101.9 | 15 | 0.8558 | 0.8404 |
| 107 | RM159 | 5 | 5.4 | 14 | 0.8929 | 0.8832 |  | 237 | RM7163 | 11 | 102.9 | 2 | 0.0765 | 0.0736 |
| 108 | RM267 | 5 | 25.0 | 12 | 0.8587 | 0.8465 |  | 238 | RM6293 | 11 | 112.4 | 6 | 0.6635 | 0.5984 |
| 109 | RM437 | 5 | 31.5 | 2 | 0.2610 | 0.2269 |  | 239 | RM224 | 11 | 117.3 | 15 | 0.8913 | 0.8814 |
| 110 | RM3193 | 5 | 36.4 | 6 | 0.7546 | 0.7229 |  | 240 | RM20 | 12 | 120.1 | 13 | 0.8123 | 0.7879 |
| 111 | RM574 | 5 | 41.0 | 7 | 0.7725 | 0.7473 |  | 241 | RM19 | 12 | 3.2 | 15 | 0.8546 | 0.8385 |
| 112 | RM249 | 5 | 50.2 | 8 | 0.7225 | 0.6786 |  | 242 | RM247 | 12 | 20.9 | 11 | 0.8216 | 0.7993 |
| 113 | RM6082 | 5 | 53.5 | 15 | 0.8528 | 0.8400 |  | 243 | RM7619 | 12 | 26.7 | 3 | 0.4923 | 0.4028 |
| 114 | RM598 | 5 | 62.7 | 6 | 0.5320 | 0.4785 |  | 244 | RM512 | 12 | 38.1 | 6 | 0.3058 | 0.2782 |
| 115 | RM473B | 5 | 78.7 | 10 | 0.8587 | 0.8425 |  | 245 | RM277 | 12 | 39.4 | 3 | 0.4193 | 0.3362 |
| 116 | RM164 | 5 | 91.4 | 8 | 0.6557 | 0.6245 |  | 246 | RM1337 | 12 | 48.2 | 9 | 0.7073 | 0.6607 |
| 117 | RM188 | 5 | 95.3 | 12 | 0.8296 | 0.8104 |  | 247 | RM511 | 12 | 51.5 | 11 | 0.8242 | 0.8022 |
| 118 | RM161 | 5 | 96.9 | 18 | 0.9044 | 0.8965 |  | 248 | RM1246 | 12 | 59.8 | 11 | 0.7140 | 0.6883 |
| 119 | RM305 | 5 | 96.9 | 7 | 0.5741 | 0.5195 |  | 249 | RM7120 | 12 | 65.3 | 6 | 0.5957 | 0.5352 |
| 120 | RM3170 | 5 | 115.4 | 14 | 0.8741 | 0.8614 |  | 250 | RM7102 | 12 | 71.8 | 10 | 0.7688 | 0.7447 |
| 121 | RM480 | 5 | 130.6 | 18 | 0.8773 | 0.8673 |  | 251 | RM309 | 12 | 73.0 | 10 | 0.8131 | 0.7887 |
| 122 | RM5818 | 5 | 144.9 | 11 | 0.8320 | 0.8120 |  | 252 | RM6869 | 12 | 75.8 | 15 | 0.8829 | 0.8718 |
| 123 | RM8109 | 6 | 1.7 | 11 | 0.7753 | 0.7463 |  | 253 | RM463 | 12 | 75.5 | 8 | 0.4294 | 0.4088 |
| 124 | RM508 | 6 | 2.3 | 15 | 0.8371 | 0.8189 |  | 254 | RM3331 | 12 | 89.5 | 9 | 0.7149 | 0.6661 |
| 125 | RM510 | 6 | 11.5 | 12 | 0.8261 | 0.8111 |  | 255 | RM270 | 12 | 91.3 | 9 | 0.7375 | 0.7002 |
| 126 | RM225 | 6 | 26.2 | 12 | 0.7552 | 0.7209 |  | 256 | RM5479 | 12 | 95.4 | 19 | 0.7239 | 0.7029 |
| 127 | RM405 | 6 | 28.6 | 6 | 0.7274 | 0.6788 |  | 257 | RM17 | 12 | 107.4 | 12 | 0.8398 | 0.8227 |
| 128 | RM50 | 6 | 32.7 | 7 | 0.6953 | 0.6629 |  | 258 | RM12 | 12 | 107.4 | 4 | 0.5341 | 0.4524 |
| 129 | RM276 | 6 | 33.5 | 13 | 0.8614 | 0.8465 |  |  | Total Alleles |  |  | 2698 |  |  |
| 130 | RM314 | 6 | 33.6 | 8 | 0.7784 | 0.7466 |  |  | Mean |  |  | 10.46 | 0.7320 | 0.7042 |

**Supplementary Table 2.** Rice materials and their membership probabilities corresponding to each subpopulation.

| **Code** | **Accessions** | **Origin** | **Q1** | **Q2** | **Q3** | **Q4** | **Q5** | **Q6** |
| --- | --- | --- | --- | --- | --- | --- | --- | --- |
| H1 | Yuedao 1 | Vietnam | 0.230 | 0.000 | 0.000 | 0.000 | 0.000 | 0.769 |
| H2 | Yuedao 2 | Vietnam | 0.276 | 0.000 | 0.000 | 0.000 | 0.000 | 0.724 |
| H3 | Yuedao 3 | Vietnam | 0.266 | 0.000 | 0.000 | 0.000 | 0.000 | 0.734 |
| H4 | Yuedao 4 | Vietnam | 0.256 | 0.000 | 0.000 | 0.000 | 0.000 | 0.744 |
| H5 | Yuedao 5 | Vietnam | 0.302 | 0.000 | 0.000 | 0.000 | 0.000 | 0.698 |
| H6 | Yuedao 6 | Vietnam | 0.275 | 0.000 | 0.000 | 0.000 | 0.000 | 0.725 |
| H7 | Yuedao 7 | Vietnam | 0.303 | 0.000 | 0.000 | 0.000 | 0.000 | 0.697 |
| H8 | Yuedao 8 | Vietnam | 0.316 | 0.000 | 0.000 | 0.000 | 0.000 | 0.684 |
| H9 | Yuedao 9 | Vietnam | 0.310 | 0.000 | 0.000 | 0.000 | 0.000 | 0.690 |
| H10 | Yuedao 10 | Vietnam | 0.297 | 0.000 | 0.000 | 0.000 | 0.000 | 0.703 |
| H11 | Yuedao 11 | Vietnam | 0.287 | 0.000 | 0.000 | 0.000 | 0.000 | 0.713 |
| H12 | Yuedao 12 | Vietnam | 0.303 | 0.000 | 0.000 | 0.000 | 0.000 | 0.696 |
| H13 | Yuedao 13 | Vietnam | 0.295 | 0.000 | 0.000 | 0.000 | 0.000 | 0.705 |
| H14 | Yuedao 14 | Vietnam | 0.299 | 0.000 | 0.000 | 0.000 | 0.000 | 0.700 |
| H15 | Yuedao 15 | Vietnam | 0.278 | 0.000 | 0.000 | 0.000 | 0.000 | 0.721 |
| H16 | Yuedao 16 | Vietnam | 0.281 | 0.000 | 0.000 | 0.000 | 0.000 | 0.718 |
| H17 | Yuedao 17 | Vietnam | 0.284 | 0.000 | 0.000 | 0.000 | 0.000 | 0.716 |
| H18 | Yuedao 18 | Vietnam | 0.999 | 0.000 | 0.000 | 0.000 | 0.000 | 0.000 |
| H19 | Yuedao 19 | Vietnam | 1.000 | 0.000 | 0.000 | 0.000 | 0.000 | 0.000 |
| H20 | Yuedao 20 | Vietnam | 1.000 | 0.000 | 0.000 | 0.000 | 0.000 | 0.000 |
| H21 | Yuedao 21 | Vietnam | 1.000 | 0.000 | 0.000 | 0.000 | 0.000 | 0.000 |
| H22 | Yuedao 22 | Vietnam | 1.000 | 0.000 | 0.000 | 0.000 | 0.000 | 0.000 |
| H23 | Yuedao 23 | Vietnam | 0.999 | 0.000 | 0.000 | 0.000 | 0.000 | 0.000 |
| H24 | Yuedao 24 | Vietnam | 1.000 | 0.000 | 0.000 | 0.000 | 0.000 | 0.000 |
| H25 | Yuedao 25 | Vietnam | 1.000 | 0.000 | 0.000 | 0.000 | 0.000 | 0.000 |
| H26 | Yuedao 26 | Vietnam | 1.000 | 0.000 | 0.000 | 0.000 | 0.000 | 0.000 |
| H27 | Yuedao 27 | Vietnam | 1.000 | 0.000 | 0.000 | 0.000 | 0.000 | 0.000 |
| H28 | Yuedao 28 | Vietnam | 1.000 | 0.000 | 0.000 | 0.000 | 0.000 | 0.000 |
| H29 | Yuedao 29 | Vietnam | 1.000 | 0.000 | 0.000 | 0.000 | 0.000 | 0.000 |
| H30 | Yuedao 30 | Vietnam | 1.000 | 0.000 | 0.000 | 0.000 | 0.000 | 0.000 |
| H31 | Yuedao 31 | Vietnam | 1.000 | 0.000 | 0.000 | 0.000 | 0.000 | 0.000 |
| H32 | Yuedao 32 | Vietnam | 1.000 | 0.000 | 0.000 | 0.000 | 0.000 | 0.000 |
| H33 | Yuedao 33 | Vietnam | 1.000 | 0.000 | 0.000 | 0.000 | 0.000 | 0.000 |
| H34 | Yuedao 34 | Vietnam | 1.000 | 0.000 | 0.000 | 0.000 | 0.000 | 0.000 |
| H35 | Yuedao 35 | Vietnam | 1.000 | 0.000 | 0.000 | 0.000 | 0.000 | 0.000 |
| H36 | Yuedao 36 | Vietnam | 1.000 | 0.000 | 0.000 | 0.000 | 0.000 | 0.000 |
| H37 | Yuedao 37 | Vietnam | 1.000 | 0.000 | 0.000 | 0.000 | 0.000 | 0.000 |
| H38 | Yuedao 38 | Vietnam | 1.000 | 0.000 | 0.000 | 0.000 | 0.000 | 0.000 |
| H39 | Yuedao 39 | Vietnam | 1.000 | 0.000 | 0.000 | 0.000 | 0.000 | 0.000 |
| H40 | Yuedao 40 | Vietnam | 1.000 | 0.000 | 0.000 | 0.000 | 0.000 | 0.000 |
| H41 | Yuedao 41 | Vietnam | 1.000 | 0.000 | 0.000 | 0.000 | 0.000 | 0.000 |
| H42 | Yuedao 42 | Vietnam | 1.000 | 0.000 | 0.000 | 0.000 | 0.000 | 0.000 |
| H43 | Yuedao 43 | Vietnam | 1.000 | 0.000 | 0.000 | 0.000 | 0.000 | 0.000 |
| H44 | Yuedao 44 | Vietnam | 1.000 | 0.000 | 0.000 | 0.000 | 0.000 | 0.000 |
| H45 | Yuedao 45 | Vietnam | 1.000 | 0.000 | 0.000 | 0.000 | 0.000 | 0.000 |
| H46 | Yuedao 46 | Vietnam | 1.000 | 0.000 | 0.000 | 0.000 | 0.000 | 0.000 |
| H47 | Yuedao 47 | Vietnam | 1.000 | 0.000 | 0.000 | 0.000 | 0.000 | 0.000 |
| H48 | Yuedao 48 | Vietnam | 0.999 | 0.000 | 0.000 | 0.000 | 0.000 | 0.000 |
| H49 | Yuedao 49 | Vietnam | 1.000 | 0.000 | 0.000 | 0.000 | 0.000 | 0.000 |
| H50 | Yuedao 50 | Vietnam | 1.000 | 0.000 | 0.000 | 0.000 | 0.000 | 0.000 |
| H51 | Yuedao 51 | Vietnam | 1.000 | 0.000 | 0.000 | 0.000 | 0.000 | 0.000 |
| H52 | Yuedao 52 | Vietnam | 1.000 | 0.000 | 0.000 | 0.000 | 0.000 | 0.000 |
| H53 | Yuedao 53 | Vietnam | 1.000 | 0.000 | 0.000 | 0.000 | 0.000 | 0.000 |
| H54 | Yuedao 54 | Vietnam | 0.590 | 0.000 | 0.000 | 0.000 | 0.000 | 0.409 |
| H55 | Yuedao 55 | Vietnam | 0.998 | 0.000 | 0.001 | 0.000 | 0.000 | 0.000 |
| H56 | Yuedao 56 | Vietnam | 0.999 | 0.000 | 0.000 | 0.000 | 0.000 | 0.000 |
| H57 | Yuedao 57 | Vietnam | 1.000 | 0.000 | 0.000 | 0.000 | 0.000 | 0.000 |
| H58 | Yuedao 58 | Vietnam | 1.000 | 0.000 | 0.000 | 0.000 | 0.000 | 0.000 |
| H59 | Yuedao 59 | Vietnam | 1.000 | 0.000 | 0.000 | 0.000 | 0.000 | 0.000 |
| H60 | Yuedao 60 | Vietnam | 1.000 | 0.000 | 0.000 | 0.000 | 0.000 | 0.000 |
| H61 | Yuedao 61 | Vietnam | 1.000 | 0.000 | 0.000 | 0.000 | 0.000 | 0.000 |
| H62 | Yuedao 62 | Vietnam | 1.000 | 0.000 | 0.000 | 0.000 | 0.000 | 0.000 |
| H63 | Yuedao 63 | Vietnam | 0.999 | 0.000 | 0.000 | 0.000 | 0.000 | 0.000 |
| H64 | Yuedao 64 | Vietnam | 0.993 | 0.001 | 0.004 | 0.000 | 0.002 | 0.001 |
| H65 | Yuedao 65 | Vietnam | 0.999 | 0.000 | 0.000 | 0.000 | 0.000 | 0.000 |
| H66 | Yuedao 66 | Vietnam | 0.991 | 0.001 | 0.005 | 0.001 | 0.001 | 0.000 |
| H67 | Yuedao 67 | Vietnam | 1.000 | 0.000 | 0.000 | 0.000 | 0.000 | 0.000 |
| H68 | Yuedao 68 | Vietnam | 1.000 | 0.000 | 0.000 | 0.000 | 0.000 | 0.000 |
| H69 | Yuedao 69 | Vietnam | 1.000 | 0.000 | 0.000 | 0.000 | 0.000 | 0.000 |
| H70 | Yuedao 70 | Vietnam | 1.000 | 0.000 | 0.000 | 0.000 | 0.000 | 0.000 |
| H71 | Yuedao 71 | Vietnam | 0.999 | 0.000 | 0.000 | 0.000 | 0.000 | 0.000 |
| H72 | Yuedao 72 | Vietnam | 1.000 | 0.000 | 0.000 | 0.000 | 0.000 | 0.000 |
| H73 | Yuedao 73 | Vietnam | 1.000 | 0.000 | 0.000 | 0.000 | 0.000 | 0.000 |
| H74 | Yuedao 74 | Vietnam | 1.000 | 0.000 | 0.000 | 0.000 | 0.000 | 0.000 |
| H75 | Yuedao 75 | Vietnam | 1.000 | 0.000 | 0.000 | 0.000 | 0.000 | 0.000 |
| H76 | Yuedao 76 | Vietnam | 1.000 | 0.000 | 0.000 | 0.000 | 0.000 | 0.000 |
| H77 | Yuedao 77 | Vietnam | 1.000 | 0.000 | 0.000 | 0.000 | 0.000 | 0.000 |
| H78 | Yuedao 78 | Vietnam | 0.987 | 0.000 | 0.005 | 0.000 | 0.007 | 0.000 |
| H79 | Yuedao 79 | Vietnam | 1.000 | 0.000 | 0.000 | 0.000 | 0.000 | 0.000 |
| H80 | Yuedao 80 | Vietnam | 1.000 | 0.000 | 0.000 | 0.000 | 0.000 | 0.000 |
| H81 | Yuedao 81 | Vietnam | 1.000 | 0.000 | 0.000 | 0.000 | 0.000 | 0.000 |
| H82 | Yuedao 82 | Vietnam | 1.000 | 0.000 | 0.000 | 0.000 | 0.000 | 0.000 |
| H83 | Yuedao 83 | Vietnam | 1.000 | 0.000 | 0.000 | 0.000 | 0.000 | 0.000 |
| H84 | Yuedao 84 | Vietnam | 0.592 | 0.000 | 0.000 | 0.000 | 0.000 | 0.408 |
| H85 | Yuedao 85 | Vietnam | 1.000 | 0.000 | 0.000 | 0.000 | 0.000 | 0.000 |
| H86 | Yuedao 86 | Vietnam | 0.983 | 0.001 | 0.010 | 0.000 | 0.005 | 0.001 |
| H87 | Yuedao 87 | Vietnam | 1.000 | 0.000 | 0.000 | 0.000 | 0.000 | 0.000 |
| H88 | Yuedao 88 | Vietnam | 1.000 | 0.000 | 0.000 | 0.000 | 0.000 | 0.000 |
| H89 | Yuedao 89 | Vietnam | 1.000 | 0.000 | 0.000 | 0.000 | 0.000 | 0.000 |
| H90 | Yuedao 90 | Vietnam | 1.000 | 0.000 | 0.000 | 0.000 | 0.000 | 0.000 |
| H91 | Yuedao 91 | Vietnam | 0.999 | 0.000 | 0.000 | 0.000 | 0.000 | 0.000 |
| H92 | Yuedao 92 | Vietnam | 1.000 | 0.000 | 0.000 | 0.000 | 0.000 | 0.000 |
| H93 | Yuedao 93 | Vietnam | 1.000 | 0.000 | 0.000 | 0.000 | 0.000 | 0.000 |
| H94 | Yuedao 94 | Vietnam | 1.000 | 0.000 | 0.000 | 0.000 | 0.000 | 0.000 |
| H95 | Yuedao 95 | Vietnam | 0.999 | 0.000 | 0.000 | 0.000 | 0.000 | 0.000 |
| H96 | Yuedao 96 | Vietnam | 0.982 | 0.000 | 0.017 | 0.000 | 0.000 | 0.000 |
| H97 | Yuedao 97 | Vietnam | 1.000 | 0.000 | 0.000 | 0.000 | 0.000 | 0.000 |
| H98 | Yuedao 98 | Vietnam | 1.000 | 0.000 | 0.000 | 0.000 | 0.000 | 0.000 |
| H99 | Yuedao 99 | Vietnam | 1.000 | 0.000 | 0.000 | 0.000 | 0.000 | 0.000 |
| H100 | Yuedao 100 | Vietnam | 1.000 | 0.000 | 0.000 | 0.000 | 0.000 | 0.000 |
| H101 | Yuedao 101 | Vietnam | 1.000 | 0.000 | 0.000 | 0.000 | 0.000 | 0.000 |
| H102 | Yuedao 102 | Vietnam | 1.000 | 0.000 | 0.000 | 0.000 | 0.000 | 0.000 |
| H103 | Yuedao 103 | Vietnam | 0.999 | 0.000 | 0.000 | 0.000 | 0.000 | 0.000 |
| H104 | Yuedao 104 | Vietnam | 1.000 | 0.000 | 0.000 | 0.000 | 0.000 | 0.000 |
| H105 | Yuedao 105 | Vietnam | 1.000 | 0.000 | 0.000 | 0.000 | 0.000 | 0.000 |
| H106 | Yuedao 106 | Vietnam | 1.000 | 0.000 | 0.000 | 0.000 | 0.000 | 0.000 |
| H107 | Yuedao 107 | Vietnam | 1.000 | 0.000 | 0.000 | 0.000 | 0.000 | 0.000 |
| H108 | Yuedao 108 | Vietnam | 0.990 | 0.000 | 0.008 | 0.000 | 0.001 | 0.000 |
| H109 | Yuedao 109 | Vietnam | 0.948 | 0.012 | 0.018 | 0.000 | 0.022 | 0.000 |
| H110 | Yuedao 110 | Vietnam | 0.999 | 0.000 | 0.000 | 0.000 | 0.000 | 0.000 |
| H111 | Yuedao 111 | Vietnam | 1.000 | 0.000 | 0.000 | 0.000 | 0.000 | 0.000 |
| H112 | Yuedao 112 | Vietnam | 1.000 | 0.000 | 0.000 | 0.000 | 0.000 | 0.000 |
| H113 | Yuedao 113 | Vietnam | 1.000 | 0.000 | 0.000 | 0.000 | 0.000 | 0.000 |
| H114 | Yuedao 114 | Vietnam | 0.490 | 0.000 | 0.001 | 0.000 | 0.509 | 0.000 |
| H115 | Yuedao 115 | Vietnam | 0.416 | 0.000 | 0.000 | 0.000 | 0.582 | 0.000 |
| H116 | Yuedao 116 | Vietnam | 0.445 | 0.000 | 0.000 | 0.000 | 0.554 | 0.000 |
| H117 | Yuedao 117 | Vietnam | 0.419 | 0.000 | 0.000 | 0.000 | 0.580 | 0.000 |
| H118 | Yuedao 118 | Vietnam | 0.393 | 0.000 | 0.000 | 0.000 | 0.607 | 0.000 |
| H119 | Yuedao 119 | Vietnam | 0.275 | 0.000 | 0.000 | 0.000 | 0.721 | 0.001 |
| H120 | Yuedao 120 | Vietnam | 0.365 | 0.000 | 0.000 | 0.000 | 0.634 | 0.000 |
| H121 | Yuedao 121 | Vietnam | 0.340 | 0.000 | 0.000 | 0.000 | 0.659 | 0.000 |
| H122 | Taijing 9 | Taibei, Tianwan | 0.000 | 0.000 | 0.000 | 0.000 | 0.000 | 0.999 |
| H123 | Taijing16xuanAC | Taibei,Tianwan | 0.000 | 0.000 | 0.000 | 0.000 | 0.000 | 0.999 |
| H124 | Taijing16xuanzi | Taibei,Tianwan | 0.000 | 0.000 | 0.000 | 0.000 | 0.000 | 0.999 |
| H125 | Diantun502xuanzao | Kunming, Yunnan | 0.000 | 0.000 | 0.000 | 0.000 | 0.000 | 0.997 |
| H126 | Nongxiang 21 | Changsha, Hunan | 0.000 | 0.000 | 0.000 | 0.000 | 0.000 | 0.999 |
| H127 | Nongxiang 25 | Changsha, Hunan | 0.000 | 0.000 | 0.000 | 0.000 | 0.000 | 0.999 |
| H128 | Nongxiang 26 | Changsha, Hunan | 0.000 | 0.000 | 0.000 | 0.000 | 0.000 | 0.999 |
| H129 | Yuzhenxiang | Changsha, Hunan | 0.000 | 0.000 | 0.000 | 0.000 | 0.000 | 0.999 |
| H130 | Xiangwanxian17 | Changsha, Hunan | 0.000 | 0.000 | 0.000 | 0.000 | 0.000 | 0.999 |
| H131 | Huanghuazhan | Changsha, Hunan | 0.002 | 0.000 | 0.000 | 0.000 | 0.000 | 0.999 |
| H132 | Fengyouwan 8hao | Changsha, Hunan | 0.000 | 0.000 | 0.000 | 0.000 | 0.000 | 0.999 |
| H133 | Yazihuang | Jinshan, Shanghai | 0.000 | 0.000 | 0.017 | 0.001 | 0.965 | 0.016 |
| H134 | Longgouzhong | Qingpu, Shanghai | 0.000 | 0.000 | 0.000 | 0.000 | 1.000 | 0.000 |
| H135 | Maijieqing | Songjiang, Shanghai | 0.000 | 0.000 | 0.999 | 0.000 | 0.000 | 0.000 |
| H136 | Shuaishaban | Songjiang, Shanghai | 0.000 | 0.000 | 0.999 | 0.000 | 0.000 | 0.000 |
| H137 | Manliuzhong | Jinshan, Shanghai | 0.000 | 0.000 | 0.999 | 0.000 | 0.000 | 0.000 |
| H138 | Tainluohuang | Changshu, Jiangsu | 0.000 | 0.000 | 1.000 | 0.000 | 0.000 | 0.000 |
| H139 | Jiucaiqing | Changshu, Jiangsu | 0.000 | 0.000 | 1.000 | 0.000 | 0.000 | 0.000 |
| H140 | Aiqidalvzhong | Jiading, Shanghai | 0.000 | 0.000 | 1.000 | 0.000 | 0.000 | 0.000 |
| H141 | Yishixing | Changshu, Jiangsu | 0.000 | 0.000 | 0.999 | 0.000 | 0.000 | 0.000 |
| H142 | Diediezhong | Qingpu, Shanghai | 0.000 | 0.000 | 0.999 | 0.000 | 0.000 | 0.000 |
| H143 | Baishuqing | Qingpu, Shanghai | 0.000 | 0.000 | 1.000 | 0.000 | 0.000 | 0.000 |
| H144 | Xiaoqingmang | Changshu, Jiangsu | 0.000 | 0.000 | 0.999 | 0.000 | 0.000 | 0.000 |
| H145 | Lvzhong | Changshu, Jiangsu | 0.000 | 0.000 | 0.999 | 0.000 | 0.000 | 0.000 |
| H146 | Jiaobaiyeqing | Songjiang, Shanghai | 0.121 | 0.001 | 0.878 | 0.000 | 0.000 | 0.000 |
| H147 | Eyingbaijingdao | Jiading, Shanghai | 0.000 | 0.000 | 1.000 | 0.000 | 0.000 | 0.000 |
| H148 | Dadaosuitou | Changshu, Jiangsu | 0.000 | 0.000 | 0.999 | 0.000 | 0.000 | 0.000 |
| H149 | Wumangyedao | Changshu, Jiangsu | 0.000 | 0.000 | 0.999 | 0.000 | 0.000 | 0.000 |
| H150 | Xiangqing | Chongming, Shanghai | 0.043 | 0.000 | 0.956 | 0.000 | 0.000 | 0.000 |
| H151 | R254 | Chongming, Shanghai | 0.000 | 0.000 | 0.221 | 0.000 | 0.000 | 0.778 |
| H152 | Datougui | Changshu, Jiangsu | 0.000 | 0.000 | 0.000 | 0.000 | 0.000 | 1.000 |
| H153 | Kuobanzhong | Qingpu, Shanghai | 0.000 | 0.000 | 0.000 | 0.000 | 0.000 | 1.000 |
| H154 | Aijiaoluganhuang | Changshu, Jiangsu | 0.000 | 0.000 | 0.000 | 0.000 | 0.000 | 1.000 |
| H155 | Jia159 | Jiaxing, Zhejiang | 0.000 | 0.000 | 0.000 | 0.000 | 0.093 | 0.906 |
| H156 | Xiushui79 | Jiaxing, Zhejiang | 0.000 | 0.000 | 0.000 | 0.000 | 0.044 | 0.948 |
| H157 | Hongbaodao | Jiaxing, Zhejiang | 0.000 | 0.000 | 1.000 | 0.000 | 0.000 | 0.000 |
| H158 | Jia45 | Jiaxing, Zhejiang | 0.000 | 0.001 | 0.000 | 0.000 | 0.998 | 0.000 |
| H159 | Hongmangshajing | Kunshan, Jiangsu | 0.109 | 0.000 | 0.002 | 0.066 | 0.821 | 0.000 |
| H160 | Wanhuangdao | Wuxian, Jiangsu | 0.000 | 0.001 | 0.001 | 0.001 | 0.996 | 0.000 |
| H161 | Guozinuo | Kunshan, Jiangsu | 0.000 | 0.000 | 0.000 | 0.000 | 0.999 | 0.000 |
| H162 | Shuijingbaidao | Wuxian, Jiangsu | 0.000 | 0.000 | 0.000 | 0.000 | 0.999 | 0.000 |
| H163 | Wumangzaodao | Changshu, Jiangsu | 0.000 | 0.000 | 0.001 | 0.000 | 0.999 | 0.000 |
| H164 | Sanbailitou | Kunshan, Jiangsu | 0.000 | 0.000 | 0.000 | 0.000 | 0.999 | 0.000 |
| H165 | Cuyingwanyangdao | Wuxi, Jiangsu | 0.000 | 0.000 | 0.000 | 0.000 | 0.999 | 0.000 |
| H166 | Yanglingdao | Wuxi, Jiangsu | 0.000 | 0.000 | 0.000 | 0.000 | 0.999 | 0.000 |
| H167 | Wanyedao | Wuxian, Jiangsu | 0.000 | 0.000 | 0.000 | 0.000 | 0.999 | 0.000 |
| H168 | Qiaobinghuang | Taicang, Jiangsu | 0.000 | 0.000 | 0.000 | 0.000 | 1.000 | 0.000 |
| H169 | Tiejingqing | Kunshan, Jiangsu | 0.000 | 0.000 | 0.000 | 0.000 | 0.999 | 0.000 |
| H170 | Xiaobaiyedao | Wuxi, Jiangsu | 0.000 | 0.000 | 0.000 | 0.000 | 0.999 | 0.000 |
| H171 | Baoxintaihuqing | Wujiang, Jiangsu | 0.051 | 0.000 | 0.002 | 0.079 | 0.868 | 0.001 |
| H172 | Jiangfeng4 | Jiangyin, Jiangsu | 0.001 | 0.000 | 0.000 | 0.000 | 0.999 | 0.000 |
| H173 | Sujing4 | Suzhou, Jiangsu | 0.000 | 0.000 | 0.000 | 0.000 | 0.999 | 0.000 |
| H174 | Aizhongluohanhuang | Changshu, Jiangsu | 0.000 | 0.000 | 0.000 | 0.000 | 1.000 | 0.000 |
| H175 | Baodao | Wuxi, Jiangsu | 0.000 | 0.000 | 0.000 | 0.000 | 1.000 | 0.000 |
| H176 | Wanmuxiqiu | Taicang, Jiangsu | 0.000 | 0.000 | 0.000 | 0.000 | 1.000 | 0.000 |
| H177 | Huangsanshi | Wujiang, Jiangsu | 0.000 | 0.000 | 0.000 | 0.000 | 1.000 | 0.000 |
| H178 | Erheidao | Wuxi, Jiangsu | 0.000 | 0.000 | 0.000 | 0.000 | 1.000 | 0.000 |
| H179 | Xiaoqingzhong | Wuxian, Jiangsu | 0.000 | 0.000 | 0.000 | 0.000 | 1.000 | 0.000 |
| H180 | Zaoguangtou | Wuxi, Jiangsu | 0.000 | 0.000 | 0.000 | 0.000 | 1.000 | 0.000 |
| H181 | Xiaoluohanhuang | Changshu, Jiangsu | 0.000 | 0.000 | 0.000 | 0.000 | 1.000 | 0.000 |
| H182 | Souzhouqing | Jiangyin, Jiangsu | 0.000 | 0.000 | 0.000 | 0.000 | 1.000 | 0.000 |
| H183 | Wanluli | Jiangyin, Jiangsu | 0.000 | 0.000 | 0.000 | 0.000 | 0.999 | 0.000 |
| H184 | Wanbaguo | Jiangyin, Jiangsu | 0.000 | 0.000 | 0.000 | 0.000 | 1.000 | 0.000 |
| H185 | Ebusinuodao | Wuxi, Jiangsu | 0.000 | 0.000 | 0.000 | 0.000 | 0.999 | 0.000 |
| H186 | Laodiegu | Wujiang, Jiangsu | 0.000 | 0.000 | 0.000 | 0.000 | 1.000 | 0.000 |
| H187 | Yefenghuang | Wujiang, Jiangsu | 0.000 | 0.000 | 0.000 | 0.000 | 1.000 | 0.000 |
| H188 | Chenjiazhong | Kunshan, Jiangsu | 0.000 | 0.000 | 0.000 | 0.000 | 1.000 | 0.000 |
| H189 | Zaoheitouhong | Wujiang, Jiangsu | 0.000 | 0.000 | 0.000 | 0.000 | 1.000 | 0.000 |
| H190 | Luohanhuang | Jiangyin, Jiangsu | 0.000 | 0.000 | 0.000 | 0.000 | 1.000 | 0.000 |
| H191 | Shiluqing | Kunshan, Jiangsu | 0.000 | 0.000 | 0.000 | 0.000 | 1.000 | 0.000 |
| H192 | Ligengqing | Yixing, Jiangsu | 0.000 | 0.000 | 0.000 | 0.000 | 1.000 | 0.000 |
| H193 | Heitouhong | Wujiang, Jiangsu | 0.000 | 0.000 | 0.000 | 0.000 | 1.000 | 0.000 |
| H194 | Laolaihong | Wuxian, Jiangsu | 0.000 | 0.000 | 0.000 | 0.000 | 1.000 | 0.000 |
| H195 | Erlibie | Wuxian, Jiangsu | 0.000 | 0.000 | 0.000 | 0.000 | 1.000 | 0.000 |
| H196 | Jinguhuang | Wujiang, Jiangsu | 0.000 | 0.000 | 0.000 | 0.000 | 1.000 | 0.000 |
| H197 | Cuganhuangdao | Wujiang, Jiangsu | 0.000 | 0.000 | 0.000 | 0.000 | 1.000 | 0.000 |
| H198 | Zaoshirihuangdao | Wuxian, Jiangsu | 0.000 | 0.000 | 0.000 | 0.000 | 1.000 | 0.000 |
| H199 | Shengtangqing | Changshu, Jiangsu | 0.000 | 0.000 | 0.000 | 0.000 | 1.000 | 0.000 |
| H200 | Xiaomandao | Wujiang, Jiangsu | 0.000 | 0.000 | 0.000 | 0.000 | 1.000 | 0.000 |
| H201 | Wanmandao | Wujiang, Jiangsu | 0.000 | 0.000 | 0.000 | 0.000 | 0.999 | 0.000 |
| H202 | Nantouzhong | Kunshan, Jiangsu | 0.000 | 0.000 | 0.000 | 0.000 | 0.999 | 0.000 |
| H203 | Daniaodao | Changshu, Jiangsu | 0.000 | 0.000 | 0.000 | 0.000 | 0.998 | 0.001 |
| H204 | Kongqueqing | Kunshan, Jiangsu | 0.005 | 0.000 | 0.043 | 0.000 | 0.560 | 0.392 |
| H205 | Kaiqing | Kunshan, Jiangsu | 0.013 | 0.000 | 0.012 | 0.000 | 0.502 | 0.473 |
| H206 | Manyedao | Kunshan, Jiangsu | 0.001 | 0.000 | 0.000 | 0.000 | 0.573 | 0.425 |
| H207 | Baikenuo | Wujiang, Jiangsu | 0.108 | 0.000 | 0.000 | 0.001 | 0.511 | 0.380 |
| H208 | Baimangnuo | Wujiang, Jiangsu | 0.001 | 0.000 | 0.012 | 0.000 | 0.576 | 0.411 |
| H209 | Xiangzhunuo | Changshu, Jiangsu | 0.001 | 0.000 | 0.001 | 0.000 | 0.577 | 0.421 |
| H210 | Yaxienuo | Wuxian, Jiangsu | 0.001 | 0.000 | 0.002 | 0.000 | 0.543 | 0.453 |
| H211 | Xianhui429 | Nanjing, Jiangsu | 0.002 | 0.000 | 0.002 | 0.000 | 0.514 | 0.482 |
| H212 | Zijianxian 3 | Nanjing, Jiangsu | 0.115 | 0.000 | 0.000 | 0.005 | 0.377 | 0.503 |
| H213 | Huangsandannuo | Wuxi, Jiangsu | 0.116 | 0.000 | 0.001 | 0.000 | 0.378 | 0.504 |
| H214 | Wuqiang | Wujin, Jiangsu | 0.000 | 0.000 | 0.000 | 0.000 | 0.983 | 0.016 |
| H215 | Wuyujing 3 | Wujin, Jiangsu | 0.000 | 0.000 | 0.000 | 0.000 | 0.990 | 0.009 |
| H216 | Xiushui 04 | Nanjing, Jiangsu | 0.000 | 0.000 | 0.000 | 0.000 | 0.997 | 0.003 |
| H217 | Zhendao 88 | Zhenjiang, Jiangsu | 0.000 | 0.000 | 0.000 | 0.000 | 0.999 | 0.000 |
| H218 | Zhendao 6 | Zhenjiang, Jiangsu | 0.000 | 0.000 | 0.000 | 0.000 | 0.999 | 0.000 |
| H219 | 6427 | Nanjing, Jiangsu | 0.000 | 0.000 | 0.000 | 0.000 | 0.999 | 0.000 |
| H220 | Zijianjingnuo | Nanjing, Jiangsu | 0.000 | 0.000 | 0.000 | 0.000 | 0.999 | 0.000 |
| H221 | Nannongjing 62401 | Nanjing, Jiangsu | 0.011 | 0.001 | 0.000 | 0.000 | 0.983 | 0.003 |
| H222 | Tongjing 109 | Nantong, Jiangsu | 0.000 | 0.000 | 0.000 | 0.000 | 0.000 | 0.999 |
| H223 | Ningjing 1 | Nanjing, Jiangsu | 0.000 | 0.000 | 0.000 | 0.000 | 0.000 | 0.999 |
| H224 | Wujing 15 | Wujin, Jiangsu | 0.000 | 0.000 | 0.000 | 0.000 | 0.000 | 0.999 |
| H225 | Wuxiangjing 14 | Wujin, Jiangsu | 0.000 | 0.000 | 0.000 | 0.000 | 0.000 | 1.000 |
| H226 | Nannongjing 003 | Nanjing, Jiangsu | 0.000 | 0.000 | 0.000 | 0.000 | 0.000 | 0.999 |
| H227 | Nannongjing 005 | Nanjing, Jiangsu | 0.000 | 0.000 | 0.000 | 0.000 | 0.000 | 0.999 |
| H228 | 5jing20 | Nanjing, Jiangsu | 0.000 | 0.000 | 0.000 | 0.000 | 0.000 | 0.999 |
| H229 | 5jing15 | Nanjing, Jiangsu | 0.000 | 0.000 | 0.000 | 0.000 | 0.000 | 0.999 |
| H230 | molingjing | Nanjing, Jiangsu | 0.000 | 0.000 | 0.000 | 0.000 | 0.000 | 0.999 |
| H231 | 5jing03 | Nanjing, Jiangsu | 0.000 | 0.000 | 0.000 | 0.000 | 0.000 | 0.999 |
| H232 | 5jing68 | Nanjing, Jiangsu | 0.000 | 0.000 | 0.000 | 0.000 | 0.000 | 0.999 |
| H233 | Zhen9424 | Zhenjiang, Jiangsu | 0.000 | 0.169 | 0.000 | 0.000 | 0.229 | 0.601 |
| H234 | Wuyujing 7 | Wujin, Jiangsu | 0.000 | 0.199 | 0.000 | 0.000 | 0.110 | 0.691 |
| H235 | Sujing 353 | Suzhou, Jiangsu | 0.000 | 0.000 | 0.000 | 0.000 | 0.000 | 0.999 |
| H236 | Xiangjing 9407 | Nanjing, Jiangsu | 0.000 | 0.000 | 0.000 | 0.000 | 0.000 | 0.999 |
| H237 | Zhongjing 212 | Nanjing, Jiangsu | 0.000 | 0.000 | 0.000 | 0.000 | 0.000 | 0.999 |
| H238 | Zhongjing 9677 | Nanjing, Jiangsu | 0.000 | 0.000 | 0.000 | 0.000 | 0.000 | 0.999 |
| H239 | Zhongjing 131 | Nanjing, Jiangsu | 0.000 | 0.000 | 0.999 | 0.000 | 0.000 | 0.000 |
| H240 | Suwujing | Wujin, Jiangsu | 0.000 | 0.000 | 0.999 | 0.000 | 0.000 | 0.000 |
| H241 | Zhongjing 438 | Nanjing, Jiangsu | 0.000 | 0.000 | 0.999 | 0.000 | 0.000 | 0.000 |
| H242 | Zijing | Nanjing, Jiangsu | 0.000 | 0.000 | 0.999 | 0.000 | 0.000 | 0.000 |
| H243 | Zhendao10 | Zhenjiang, Jiangsu | 0.000 | 0.000 | 0.999 | 0.000 | 0.001 | 0.000 |
| H244 | Xishihuang | Wuxian, Jiangsu | 0.000 | 0.000 | 0.999 | 0.000 | 0.000 | 0.000 |
| H245 | Daliangdao | Wuxi, Jiangsu | 0.000 | 0.000 | 0.999 | 0.000 | 0.000 | 0.000 |
| H246 | Heizuidao | Changshu, Jiangsu | 0.000 | 0.000 | 0.999 | 0.000 | 0.000 | 0.000 |
| H247 | Xiaohuangdao | Wuxian, Jiangsu | 0.000 | 0.000 | 0.999 | 0.000 | 0.000 | 0.000 |
| H248 | Fenghuangdao | Changshu, Jiangsu | 0.000 | 0.000 | 0.999 | 0.000 | 0.000 | 0.000 |
| H249 | Jijingdao | Wujiang, Jiangsu | 0.000 | 0.000 | 0.999 | 0.000 | 0.000 | 0.000 |
| H250 | Zhongsuyangzhogndao | Wuxi, Jiangsu | 0.000 | 0.000 | 0.999 | 0.000 | 0.000 | 0.000 |
| H251 | Duiguzhong | Wujiang, Jiangsu | 0.000 | 0.000 | 0.999 | 0.000 | 0.000 | 0.000 |
| H252 | Niumaohuang | Taicang, Jiangsu | 0.000 | 0.000 | 0.999 | 0.000 | 0.000 | 0.000 |
| H253 | Wanheitouhong | Wujiang, Jiangsu | 0.000 | 0.000 | 0.999 | 0.000 | 0.000 | 0.000 |
| H254 | Taihuqing | Kunshan, Jiangsu | 0.000 | 0.000 | 0.999 | 0.000 | 0.000 | 0.000 |
| H255 | Yilimang | Changshu, Jiangsu | 0.000 | 0.000 | 0.999 | 0.000 | 0.000 | 0.000 |
| H256 | Wuqitou | Wujin, Jiangsu | 0.000 | 0.000 | 0.999 | 0.000 | 0.000 | 0.000 |
| H257 | Jiaoaiheitouhong | Wujiang, Jiangsu | 0.000 | 0.000 | 0.999 | 0.000 | 0.000 | 0.000 |
| H258 | Laowusi | Wujiang, Jiangsu | 0.000 | 0.000 | 1.000 | 0.000 | 0.000 | 0.000 |
| H259 | Lujingqing | Wujiang, Jiangsu | 0.000 | 0.000 | 1.000 | 0.000 | 0.000 | 0.000 |
| H260 | Gaoliangqing | Wujiang, Jiangsu | 0.000 | 0.000 | 0.999 | 0.000 | 0.000 | 0.000 |
| H261 | Heizhong | Wuxian, Jiangsu | 0.000 | 0.000 | 0.999 | 0.000 | 0.000 | 0.000 |
| H262 | Louhanbai | Kunshan, Jiangsu | 0.000 | 0.000 | 0.999 | 0.000 | 0.000 | 0.000 |
| H263 | Xueliqing | Wuxi, Jiangsu | 0.000 | 0.000 | 0.999 | 0.000 | 0.000 | 0.000 |
| H264 | Juhuahuang | Wuxi, Jiangsu | 0.000 | 0.000 | 0.999 | 0.000 | 0.000 | 0.000 |
| H265 | Changzijingyedao | Wuxian, Jiangsu | 0.000 | 0.000 | 1.000 | 0.000 | 0.000 | 0.000 |
| H266 | Gaidaoqing | Wujiang, Jiangsu | 0.000 | 0.000 | 1.000 | 0.000 | 0.000 | 0.000 |
| H267 | Dingzhuangdao | Wuxi, Jiangsu | 0.000 | 0.000 | 1.000 | 0.000 | 0.000 | 0.000 |
| H268 | Xuetangzhong | Jiangyin, Jiangsu | 0.000 | 0.000 | 1.000 | 0.000 | 0.000 | 0.000 |
| H269 | Guanbaidan | Wujiang, Jiangsu | 0.000 | 0.000 | 1.000 | 0.000 | 0.000 | 0.000 |
| H270 | Tiegandao | Wujiang, Jiangsu | 0.000 | 0.000 | 0.999 | 0.000 | 0.000 | 0.000 |
| H271 | Juziguang | Wuxi, Jiangsu | 0.077 | 0.000 | 0.922 | 0.000 | 0.000 | 0.000 |
| H272 | Yebaidao | Taicang, Jiangsu | 0.000 | 0.000 | 0.999 | 0.000 | 0.000 | 0.000 |
| H273 | Daheitouhong | Wujiang, Jiangsu | 0.000 | 0.000 | 0.999 | 0.000 | 0.000 | 0.000 |
| H274 | Baigedao | Wuxian, Jiangsu | 0.000 | 0.000 | 0.999 | 0.000 | 0.000 | 0.000 |
| H275 | Puxidadaotou | Wujiang, Jiangsu | 0.000 | 0.000 | 0.999 | 0.000 | 0.000 | 0.000 |
| H276 | Yangdao | Wujiang, Jiangsu | 0.000 | 0.000 | 0.999 | 0.000 | 0.000 | 0.000 |
| H277 | Yanhongdao | Wujiang, Jiangsu | 0.000 | 0.000 | 0.848 | 0.000 | 0.000 | 0.152 |
| H278 | Baikewandao | Wuxi, Jiangsu | 0.000 | 0.000 | 0.999 | 0.000 | 0.000 | 0.000 |
| H279 | Aiguodadaotou | Wujiang, Jiangsu | 0.000 | 0.000 | 1.000 | 0.000 | 0.000 | 0.000 |
| H280 | Sishitou | Wuxian, Jiangsu | 0.000 | 0.000 | 1.000 | 0.000 | 0.000 | 0.000 |
| H281 | Jiuxiaozhong | Wujiang, Jiangsu | 0.000 | 0.000 | 0.999 | 0.000 | 0.000 | 0.000 |
| H282 | Chushuhuang | Wuxian, Jiangsu | 0.000 | 0.000 | 1.000 | 0.000 | 0.000 | 0.000 |
| H283 | Qianjindao | Wujiang, Jiangsu | 0.000 | 0.000 | 0.999 | 0.000 | 0.000 | 0.000 |
| H284 | Qijiangqing | Kunshan, Jiangsu | 0.000 | 0.000 | 0.999 | 0.000 | 0.000 | 0.000 |
| H285 | Feilaifeng | Wuxi, Jiangsu | 0.000 | 0.000 | 0.999 | 0.000 | 0.000 | 0.000 |
| H286 | Kejia6 | Kunshan, Jiangsu | 0.000 | 0.000 | 1.000 | 0.000 | 0.000 | 0.000 |
| H287 | Lamujia | Kunshan, Jiangsu | 0.000 | 0.000 | 0.999 | 0.000 | 0.000 | 0.000 |
| H288 | Haonuopie | Kunshan, Jiangsu | 0.000 | 0.000 | 0.999 | 0.000 | 0.000 | 0.000 |
| H289 | Xianggu | Wujiang, Jiangsu | 0.000 | 0.000 | 1.000 | 0.000 | 0.000 | 0.000 |
| H290 | Shuangchengnuo | Wujiang, Jiangsu | 0.000 | 0.000 | 1.000 | 0.000 | 0.000 | 0.000 |
| H291 | Qiutiandaxiedao | Wujiang, Jiangsu | 0.000 | 0.000 | 1.000 | 0.000 | 0.000 | 0.000 |
| H292 | Qiyunuo10 | Wujiang, Jiangsu | 0.000 | 0.000 | 0.999 | 0.000 | 0.000 | 0.000 |
| H293 | Wunuo1 | Wujiang, Jiangsu | 0.000 | 0.000 | 0.999 | 0.000 | 0.000 | 0.000 |
| H294 | Jianongnuo2 | Wujiang, Jiangsu | 0.000 | 0.000 | 0.999 | 0.000 | 0.000 | 0.000 |
| H295 | Hongnong5 | Wujiang, Jiangsu | 0.001 | 0.000 | 0.999 | 0.000 | 0.000 | 0.000 |
| H296 | Nonglinnuo4 | Wujiang, Jiangsu | 0.000 | 0.000 | 0.999 | 0.000 | 0.000 | 0.000 |
| H297 | Xiangnuodao | Wuxian, Jiangsu | 0.000 | 0.000 | 0.999 | 0.000 | 0.000 | 0.000 |
| H298 | Luchaihong | Wujiang, Jiangsu | 0.002 | 0.000 | 0.998 | 0.000 | 0.000 | 0.000 |
| H299 | Cungu | Wujiang, Jiangsu | 0.037 | 0.000 | 0.962 | 0.000 | 0.000 | 0.000 |
| H300 | Katena | Wujiang, Jiangsu | 0.002 | 0.000 | 0.998 | 0.000 | 0.000 | 0.000 |
| H301 | Guanchanuo | Wujiang, Jiangsu | 0.003 | 0.000 | 0.996 | 0.000 | 0.001 | 0.000 |
| H302 | Kuihuanuo | Wuxian, Jiangsu | 0.069 | 0.000 | 0.930 | 0.000 | 0.000 | 0.000 |
| H303 | Suyunuo | Wuxian, Jiangsu | 0.000 | 0.000 | 0.999 | 0.000 | 0.000 | 0.000 |
| H304 | Hongjiaozhan | Wuxian, Jiangsu | 0.114 | 0.000 | 0.885 | 0.001 | 0.000 | 0.000 |
| H305 | Haobuka | Wuxian, Jiangsu | 0.158 | 0.001 | 0.840 | 0.000 | 0.002 | 0.000 |
| H306 | Chuyanghan 32 | Wuxian, Jiangsu | 0.148 | 0.002 | 0.846 | 0.000 | 0.003 | 0.000 |
| H307 | Liban 1 | Wuxian, Jiangsu | 0.116 | 0.001 | 0.882 | 0.000 | 0.000 | 0.000 |
| H308 | Kunnong 8 | Kunshan, Jiangsu | 0.001 | 0.002 | 0.996 | 0.000 | 0.001 | 0.001 |
| H309 | Guihuahuang | Nanjing, Jiangsu | 0.000 | 0.000 | 0.999 | 0.000 | 0.000 | 0.000 |
| H310 | Zhoujiazhong | Wujiang, Jiangsu | 0.000 | 0.000 | 0.999 | 0.000 | 0.000 | 0.000 |
| H311 | Xiaofenghuang | Wuxian, Jiangsu | 0.000 | 0.000 | 0.999 | 0.000 | 0.000 | 0.000 |
| H312 | Xiangjingdao | Wuxian, Jiangsu | 0.000 | 0.000 | 0.999 | 0.000 | 0.000 | 0.000 |
| H313 | Huizao | Wujiang, Jiangsu | 0.000 | 0.000 | 0.999 | 0.000 | 0.000 | 0.000 |
| H314 | Yingtoudao | Kunshan, Jiangsu | 0.000 | 0.000 | 0.999 | 0.000 | 0.000 | 0.000 |
| H315 | Changdaotou | Wujiang, Jiangsu | 0.000 | 0.000 | 0.999 | 0.000 | 0.000 | 0.000 |
| H316 | Yangmiaozhong | Wujiang, Jiangsu | 0.000 | 0.000 | 1.000 | 0.000 | 0.000 | 0.000 |
| H317 | Maoguangdao | Wuxian, Jiangsu | 0.000 | 0.000 | 1.000 | 0.000 | 0.000 | 0.000 |
| H318 | Dazhongdao | Wujiang, Jiangsu | 0.000 | 0.000 | 1.000 | 0.000 | 0.000 | 0.000 |
| H319 | Sanxiadao | Wuxi, Jiangsu | 0.000 | 0.000 | 0.999 | 0.000 | 0.000 | 0.000 |
| H320 | Hongganlizhihong | Wujiang, Jiangsu | 0.000 | 0.000 | 1.000 | 0.000 | 0.000 | 0.000 |
| H321 | Wuxidao | Changshu, Jiangsu | 0.000 | 0.000 | 0.999 | 0.000 | 0.000 | 0.000 |
| H322 | Wanzhongqiu | Wuxian, Jiangsu | 0.000 | 0.000 | 1.000 | 0.000 | 0.000 | 0.000 |
| H323 | Fengjingdao | Wuxian, Jiangsu | 0.000 | 0.000 | 1.000 | 0.000 | 0.000 | 0.000 |
| H324 | Cuganlizhihong | Wuxian, Jiangsu | 0.000 | 0.000 | 1.000 | 0.000 | 0.000 | 0.000 |
| H325 | Chiguwandao | Wujiang, Jiangsu | 0.000 | 0.000 | 0.999 | 0.000 | 0.000 | 0.000 |
| H326 | Chiguhong | Wujiang, Jiangsu | 0.002 | 0.000 | 0.998 | 0.000 | 0.000 | 0.000 |
| H327 | Fanluoqing | Kunshan, Jiangsu | 0.000 | 0.000 | 0.999 | 0.000 | 0.000 | 0.000 |
| H328 | Zaoyedao | Kunshan, Jiangsu | 0.000 | 0.000 | 1.000 | 0.000 | 0.000 | 0.000 |
| H329 | Baidiegu | Wujiang, Jiangsu | 0.000 | 0.000 | 0.999 | 0.000 | 0.000 | 0.000 |
| H330 | Wangjiadao | Wujiang, Jiangsu | 0.000 | 0.000 | 0.999 | 0.000 | 0.000 | 0.000 |
| H331 | Jiangyinzhong | Jiangyin, Jiangsu | 0.000 | 0.000 | 0.999 | 0.000 | 0.000 | 0.000 |
| H332 | Tiekewanguangtou | Wujin, Jiangsu | 0.000 | 0.000 | 0.999 | 0.000 | 0.000 | 0.000 |
| H333 | Tiekedao | Wujin, Jiangsu | 0.000 | 0.000 | 1.000 | 0.000 | 0.000 | 0.000 |
| H334 | Aibaidao | Wujiang, Jiangsu | 0.000 | 0.000 | 0.999 | 0.000 | 0.000 | 0.000 |
| H335 | Xiepihuang | Taicang, Jiangsu | 0.000 | 0.000 | 1.000 | 0.000 | 0.000 | 0.000 |
| H336 | Xiaobaidao | Wuxian, Jiangsu | 0.000 | 0.000 | 0.999 | 0.000 | 0.000 | 0.000 |
| H337 | Baishidao | Taicang, Jiangsu | 0.000 | 0.000 | 1.000 | 0.000 | 0.000 | 0.000 |
| H338 | Manbaidao | Wujiang, Jiangsu | 0.000 | 0.000 | 0.999 | 0.000 | 0.000 | 0.000 |
| H339 | Guangtouluhuabai | Wuxi, Jiangsu | 0.000 | 0.000 | 1.000 | 0.000 | 0.000 | 0.000 |
| H340 | Hongmangjing | Kunshan, Jiangsu | 0.000 | 0.000 | 1.000 | 0.000 | 0.000 | 0.000 |
| H341 | Luhuabai | Wuxian, Jiangsu | 0.000 | 0.000 | 0.999 | 0.000 | 0.000 | 0.000 |
| H342 | Haidongqing | Kunshan, Jiangsu | 0.000 | 0.000 | 0.999 | 0.000 | 0.000 | 0.000 |
| H343 | Shenlenuo | Kunshan, Jiangsu | 0.000 | 0.000 | 0.999 | 0.000 | 0.000 | 0.000 |
| H344 | Malaihong | Nanjing, Jiangsu | 0.174 | 0.000 | 0.825 | 0.000 | 0.000 | 0.000 |
| H345 | Jiangyinnuo | Jiangyin, Jiangsu | 0.000 | 0.000 | 0.205 | 0.000 | 0.000 | 0.794 |
| H346 | Jinggunuo | Wuxi, Jiangsu | 0.000 | 0.000 | 0.224 | 0.000 | 0.000 | 0.775 |
| H347 | Shanhonggu | Wujiang, Jiangsu | 0.000 | 0.000 | 0.207 | 0.000 | 0.000 | 0.792 |
| H348 | Wanshengmaohuang | Wuxi, Jiangsu | 0.000 | 0.000 | 0.239 | 0.000 | 0.000 | 0.761 |
| H349 | Wanyangdao | Wuxian, Jiangsu | 0.000 | 0.000 | 0.234 | 0.000 | 0.000 | 0.765 |
| H350 | Aidazhong | Wujiang, Jiangsu | 0.000 | 0.000 | 0.255 | 0.000 | 0.000 | 0.744 |
| H351 | Jijiaohong | Wuxian, Jiangsu | 0.000 | 0.000 | 0.214 | 0.000 | 0.000 | 0.785 |
| H352 | Toulaizhong | Wujiang, Jiangsu | 0.000 | 0.000 | 0.000 | 0.000 | 0.000 | 1.000 |
| H353 | Huakenuo | Wujiang, Jiangsu | 0.000 | 0.000 | 0.000 | 0.000 | 0.000 | 1.000 |
| H354 | Toudengyishixing | Kunshan, Jiangsu | 0.000 | 0.000 | 0.000 | 0.000 | 0.000 | 1.000 |
| H355 | Maozitou | Wujiang, Jiangsu | 0.000 | 0.000 | 0.000 | 0.000 | 0.000 | 1.000 |
| H356 | Zaonuodao | Wujiang, Jiangsu | 0.000 | 0.000 | 0.000 | 0.000 | 0.000 | 1.000 |
| H357 | Kangzhounuo | Wujiang, Jiangsu | 0.000 | 0.000 | 0.000 | 0.000 | 0.000 | 1.000 |
| H358 | Yangzhongdao | Wujiang, Jiangsu | 0.000 | 0.000 | 0.000 | 0.000 | 0.000 | 1.000 |
| H359 | Huangkewanguangtou | Wujin, Jiangsu | 0.000 | 0.000 | 0.000 | 0.000 | 0.000 | 1.000 |
| H360 | Tiehanyishixing | Wuxi, Jiangsu | 0.000 | 0.000 | 0.000 | 0.000 | 0.000 | 1.000 |
| H361 | Buxienuo | kunshan, Jiangsu | 0.000 | 0.000 | 0.000 | 0.000 | 0.000 | 1.000 |
| H362 | Nannongjing3786 | Nanjing, Jiangsu | 0.001 | 0.000 | 0.000 | 0.000 | 0.998 | 0.000 |
| H363 | 24248 | Nanjing, Jiangsu | 0.000 | 0.001 | 0.000 | 0.000 | 0.998 | 0.000 |
| H364 | Nannongjing 4004 | Nanjing, Jiangsu | 0.000 | 0.001 | 0.000 | 0.000 | 0.998 | 0.000 |
| H365 | Nannongjing 4016 | Nanjing, Jiangsu | 0.000 | 0.001 | 0.000 | 0.000 | 0.998 | 0.000 |
| H366 | Zijianwujing | Nanjing, Jiangsu | 0.000 | 0.000 | 0.000 | 0.000 | 0.999 | 0.000 |
| H367 | Ningjing 2 | Nanjing, Jiangsu | 0.000 | 0.000 | 0.000 | 0.000 | 0.999 | 0.000 |
| H368 | Wuxiang 99-8 | Wujin, Jiangsu | 0.000 | 0.000 | 0.000 | 0.000 | 0.998 | 0.000 |
| H369 | Wuyunjing 8 | Wujin, Jiangsu | 0.000 | 0.000 | 0.000 | 0.000 | 0.999 | 0.000 |
| H370 | Zhendao 99 | Zhenjiang, Jiangsu | 0.000 | 0.000 | 0.000 | 0.000 | 0.999 | 0.000 |
| H371 | Nanjing42 | Nanjing, Jiangsu | 0.000 | 0.000 | 0.000 | 0.000 | 0.999 | 0.000 |
| H372 | Wuyunjing21 | Wujin, Jiangsu | 0.000 | 0.000 | 0.000 | 0.000 | 0.999 | 0.000 |
| H373 | Heimixiandao | Nanjing, Jiangsu | 0.000 | 0.000 | 0.999 | 0.000 | 0.000 | 0.000 |
| H374 | Heimijingdao | Nanjing, Jiangsu | 0.000 | 0.000 | 1.000 | 0.000 | 0.000 | 0.000 |
| H375 | Zidao | Nanjing, Jiangsu | 0.000 | 0.000 | 1.000 | 0.000 | 0.000 | 0.000 |
| H376 | Zacaodao | Nanjing, Jiangsu | 0.000 | 0.000 | 1.000 | 0.000 | 0.000 | 0.000 |
| H377 | Youmang 429 | Nanjing, Jiangsu | 0.000 | 0.000 | 1.000 | 0.000 | 0.000 | 0.000 |
| H378 | Wanqu 429 | Nanjing, Jiangsu | 0.000 | 0.000 | 1.000 | 0.000 | 0.000 | 0.000 |
| H379 | Zhili 429 | Nanjing, Jiangsu | 0.000 | 0.000 | 1.000 | 0.000 | 0.000 | 0.000 |
| H380 | Ningjinghui096 | Nanjing, Jiangsu | 0.000 | 0.000 | 1.000 | 0.000 | 0.000 | 0.000 |
| H381 | Ningjinghui145 | Nanjing, Jiangsu | 0.000 | 0.000 | 1.000 | 0.000 | 0.000 | 0.000 |
| H382 | Ningjinghui166 | Nanjing, Jiangsu | 0.000 | 0.000 | 0.999 | 0.000 | 0.000 | 0.000 |
| H383 | Ningjinghui208 | Nanjing, Jiangsu | 0.000 | 0.000 | 0.999 | 0.000 | 0.000 | 0.000 |
| H384 | Ningjinghui210 | Nanjing, Jiangsu | 0.000 | 0.000 | 0.999 | 0.000 | 0.000 | 0.000 |
| H385 | Ningjinghui237 | Nanjing, Jiangsu | 0.000 | 0.000 | 0.999 | 0.000 | 0.000 | 0.000 |
| H386 | Ningjinghui246 | Nanjing, Jiangsu | 0.000 | 0.000 | 0.999 | 0.000 | 0.000 | 0.000 |
| H387 | Ningjinghui260 | Nanjing, Jiangsu | 0.000 | 0.000 | 0.999 | 0.000 | 0.000 | 0.000 |
| H388 | Ningjinghui286 | Nanjing, Jiangsu | 0.000 | 0.000 | 0.999 | 0.000 | 0.000 | 0.000 |
| H389 | Ningjinghui290 | Nanjing, Jiangsu | 0.000 | 0.000 | 0.999 | 0.000 | 0.000 | 0.000 |
| H390 | Ningjinghui292 | Nanjing, Jiangsu | 0.000 | 0.000 | 0.999 | 0.000 | 0.000 | 0.000 |
| H391 | Ningjinghui293 | Nanjing, Jiangsu | 0.000 | 0.000 | 1.000 | 0.000 | 0.000 | 0.000 |
| H392 | Ningjinghui296 | Nanjing, Jiangsu | 0.000 | 0.000 | 1.000 | 0.000 | 0.000 | 0.000 |
| H393 | Ningjinghui298 | Nanjing, Jiangsu | 0.000 | 0.000 | 1.000 | 0.000 | 0.000 | 0.000 |
| H394 | Ningjinghui338 | Nanjing, Jiangsu | 0.000 | 0.000 | 1.000 | 0.000 | 0.000 | 0.000 |
| H395 | Hongyin1009 | Nanjing, Jiangsu | 0.000 | 0.000 | 1.000 | 0.000 | 0.000 | 0.000 |
| H396 | Hongyin1010 | Nanjing, Jiangsu | 0.000 | 0.000 | 1.000 | 0.000 | 0.000 | 0.000 |
| H397 | Hongyin1011 | Nanjing, Jiangsu | 0.000 | 0.000 | 1.000 | 0.000 | 0.000 | 0.000 |
| H398 | Hongyin1012 | Nanjing, Jiangsu | 0.000 | 0.000 | 1.000 | 0.000 | 0.000 | 0.000 |
| H399 | Nannongjing1R | Nanjing, Jiangsu | 0.000 | 0.000 | 1.000 | 0.000 | 0.000 | 0.000 |
| H400 | Nannongjing2R | Nanjing, Jiangsu | 0.000 | 0.000 | 1.000 | 0.000 | 0.000 | 0.000 |
| H401 | Nannongjing3R | Nanjing, Jiangsu | 0.000 | 0.000 | 1.000 | 0.000 | 0.000 | 0.000 |
| H402 | Sidao10 | Siyang, Jiangsu | 0.000 | 0.000 | 0.000 | 0.993 | 0.006 | 0.000 |
| H403 | Yangdao6 | Yangzhou, Jiangsu | 0.001 | 0.000 | 0.000 | 0.999 | 0.000 | 0.000 |
| H404 | Xudao 3hao | Xuzhou, Jiangsu | 0.000 | 0.000 | 0.000 | 0.999 | 0.000 | 0.000 |
| H405 | Xudao 4hao | Xuzhou, Jiangsu | 0.000 | 0.000 | 0.000 | 0.999 | 0.000 | 0.000 |
| H406 | Xudao 5hao | Xuzhou, Jiangsu | 0.000 | 0.000 | 0.000 | 0.999 | 0.000 | 0.000 |
| H407 | Huaidao 9hao | Huaian, Jiangsu | 0.000 | 0.000 | 0.000 | 0.999 | 0.000 | 0.000 |
| H408 | Yandao 6hao | Yancheng, Jiangsu | 0.000 | 0.000 | 0.000 | 0.999 | 0.000 | 0.000 |
| H409 | Yangguang 200 | Lianyungang, Jiangsu | 0.000 | 0.000 | 0.000 | 0.999 | 0.000 | 0.000 |
| H410 | Lianjing 2 | Lianyungang, Jiangsu | 0.000 | 0.000 | 0.000 | 0.999 | 0.000 | 0.000 |
| H411 | Yanjing 8 | Yancheng, Jiangsu | 0.000 | 0.000 | 0.000 | 0.999 | 0.000 | 0.000 |
| H412 | Huaidao 11 | Huaian, Jiangsu | 0.000 | 0.000 | 0.000 | 0.999 | 0.000 | 0.000 |
| H413 | Huaidao 8 | Huaian, Jiangsu | 0.000 | 0.000 | 0.000 | 0.999 | 0.000 | 0.000 |
| H414 | Xudao2 | Xuzhou, Jiangsu | 0.001 | 0.000 | 0.000 | 0.998 | 0.000 | 0.000 |
| H415 | Yanjing9 | Yancheng, Jiangsu | 0.000 | 0.000 | 0.000 | 0.999 | 0.000 | 0.000 |
| H416 | Yangfujing4901 | Yangzhou, Jiangsu | 0.000 | 0.000 | 0.000 | 0.999 | 0.000 | 0.000 |
| H417 | Liyangxiaohongdao | Liyang, Jiangsu | 0.000 | 0.000 | 0.000 | 0.999 | 0.000 | 0.000 |
| H418 | Zaijinjing | Huaian, Jiangsu | 0.000 | 0.000 | 0.000 | 0.999 | 0.000 | 0.000 |
| H419 | Huaidao5hao | Huaian, Jiangsu | 0.000 | 0.000 | 0.000 | 0.999 | 0.000 | 0.000 |
| H420 | Yandao9 | Yancheng, Jiangsu | 0.000 | 0.000 | 0.000 | 0.999 | 0.000 | 0.000 |
| H421 | Lianjing4 | Lianyungang, Jiangsu | 0.000 | 0.000 | 0.000 | 0.999 | 0.000 | 0.000 |
| H422 | Huajing5 | Huaibei, Jiangsu | 0.000 | 0.000 | 0.000 | 0.999 | 0.000 | 0.000 |
| H423 | Huajing6 | Huaibei, Jiangsu | 0.000 | 0.000 | 0.000 | 0.999 | 0.000 | 0.000 |
| H424 | Yangfujing7 | Lixiahe, Jiangsu | 0.000 | 0.000 | 0.000 | 0.999 | 0.000 | 0.000 |
| H425 | Yangfujing8 | Lixiahe, Jiangsu | 0.000 | 0.000 | 0.000 | 0.999 | 0.000 | 0.000 |
| H426 | Lianjing9823 | Lianyungang, Jiangsu | 0.000 | 0.000 | 0.000 | 0.999 | 0.000 | 0.000 |
| H427 | Huifeng1 | Yancheng, Jiangsu | 0.000 | 0.000 | 0.000 | 0.999 | 0.000 | 0.000 |
| H428 | Huifeng2 | Yancheng, Jiangsu | 0.000 | 0.000 | 0.000 | 0.999 | 0.000 | 0.000 |
| H429 | Yandao8 | Yancheng, Jiangsu | 0.000 | 0.000 | 0.000 | 0.999 | 0.000 | 0.000 |
| H430 | Dongzheng1640 | Hongze, Jiangsu | 0.000 | 0.000 | 0.000 | 1.000 | 0.000 | 0.000 |
| H431 | Dongzhengwuyujing21 | Hongze, Jiangsu | 0.000 | 0.000 | 0.000 | 1.000 | 0.000 | 0.000 |
| H432 | 9311 | Yangzhou, Jiangsu | 0.000 | 0.000 | 0.000 | 1.000 | 0.000 | 0.000 |
| H433 | Si4029 | Sihong, Jiangsu | 0.000 | 0.000 | 0.000 | 1.000 | 0.000 | 0.000 |
| H434 | Si4031 | Sihong, Jiangsu | 0.000 | 0.000 | 0.000 | 1.000 | 0.000 | 0.000 |
| H435 | Si4033 | Sihong, Jiangsu | 0.000 | 0.000 | 0.000 | 1.000 | 0.000 | 0.000 |
| H436 | Si4039 | Sihong, Jiangsu | 0.000 | 0.000 | 0.000 | 1.000 | 0.000 | 0.000 |
| H437 | Si4040 | Sihong, Jiangsu | 0.000 | 0.000 | 0.000 | 1.000 | 0.000 | 0.000 |
| H438 | Si4041 | Sihong, Jiangsu | 0.000 | 0.000 | 0.000 | 1.000 | 0.000 | 0.000 |
| H439 | Si4049 | Sihong, Jiangsu | 0.000 | 0.000 | 0.000 | 1.000 | 0.000 | 0.000 |
| H440 | Si4079 | Sihong, Jiangsu | 0.000 | 0.000 | 0.000 | 1.000 | 0.000 | 0.000 |
| H441 | Si4081 | Sihong, Jiangsu | 0.000 | 0.000 | 0.000 | 1.000 | 0.000 | 0.000 |
| H442 | Si4082 | Sihong, Jiangsu | 0.000 | 0.000 | 0.000 | 1.000 | 0.000 | 0.000 |
| H443 | Si4139 | Sihong, Jiangsu | 0.000 | 0.000 | 0.000 | 1.000 | 0.000 | 0.000 |
| H444 | Si4152 | Sihong, Jiangsu | 0.000 | 0.000 | 0.000 | 1.000 | 0.000 | 0.000 |
| H445 | Si4161 | Sihong, Jiangsu | 0.000 | 0.000 | 0.000 | 1.000 | 0.000 | 0.000 |
| H446 | Si4229 | Sihong, Jiangsu | 0.000 | 0.000 | 0.000 | 1.000 | 0.000 | 0.000 |
| H447 | Si4230 | Sihong, Jiangsu | 0.000 | 0.000 | 0.000 | 1.000 | 0.000 | 0.000 |
| H448 | Si4251 | Sihong, Jiangsu | 0.000 | 0.000 | 0.000 | 1.000 | 0.000 | 0.000 |
| H449 | Si4252 | Sihong, Jiangsu | 0.000 | 0.000 | 0.000 | 1.000 | 0.000 | 0.000 |
| H450 | Si4259 | Sihong, Jiangsu | 0.000 | 0.000 | 0.000 | 1.000 | 0.000 | 0.000 |
| H451 | Si4263 | Sihong, Jiangsu | 0.000 | 0.000 | 0.000 | 1.000 | 0.000 | 0.000 |
| H452 | Si4280 | Sihong, Jiangsu | 0.000 | 0.000 | 0.000 | 1.000 | 0.000 | 0.000 |
| H453 | Si4330 | Sihong, Jiangsu | 0.000 | 0.000 | 0.000 | 0.999 | 0.000 | 0.000 |
| H454 | Si4360 | Sihong, Jiangsu | 0.000 | 0.000 | 0.000 | 0.999 | 0.000 | 0.000 |
| H455 | Si4364 | Sihong, Jiangsu | 0.000 | 0.000 | 0.000 | 0.999 | 0.000 | 0.000 |
| H456 | Digudao | Sihong, Jiangsu | 0.000 | 0.000 | 0.000 | 0.999 | 0.000 | 0.000 |
| H457 | Si4385 | Sihong, Jiangsu | 0.000 | 0.000 | 0.000 | 0.999 | 0.000 | 0.000 |
| H458 | Si4386 | Sihong, Jiangsu | 0.000 | 0.000 | 0.000 | 0.999 | 0.000 | 0.000 |
| H459 | Sihao4141 | Sihong, Jiangsu | 0.000 | 0.000 | 0.000 | 0.999 | 0.000 | 0.000 |
| H460 | Cbao | Hefei, Anhui | 0.000 | 0.000 | 0.001 | 0.000 | 0.049 | 0.949 |
| H461 | Wandao68 | Hefei, Anhui | 0.000 | 0.000 | 0.999 | 0.000 | 0.000 | 0.000 |
| H462 | Jingnuo330 | Hefei, Anhui | 0.000 | 0.000 | 0.258 | 0.000 | 0.000 | 0.741 |
| H463 | Fuyu3 | Yuexi, Anhui | 0.142 | 0.000 | 0.117 | 0.000 | 0.000 | 0.740 |
| H464 | Wanjingnuo | Hefei, Anhui | 0.000 | 0.000 | 0.000 | 0.000 | 0.000 | 1.000 |
| H465 | Fuxiang1 | Yuexi, Anhui | 0.000 | 0.000 | 0.000 | 0.000 | 0.000 | 1.000 |
| H466 | Zhengdao18 | Zhenzhou, Henan | 0.000 | 0.000 | 0.000 | 0.999 | 0.001 | 0.000 |
| H467 | Yujing6 | Zhenzhou, Henan | 0.000 | 0.000 | 0.000 | 0.999 | 0.000 | 0.000 |
| H468 | Zhenghan2 | Zhengzhou, Henan | 0.000 | 0.000 | 0.000 | 0.999 | 0.000 | 0.000 |
| H469 | Zhenghan 6 | Zhengzhou, Henan | 0.000 | 0.000 | 0.000 | 0.999 | 0.000 | 0.000 |
| H470 | Shengdao808 | Jiaxiang, Shandong | 0.000 | 0.000 | 0.000 | 0.999 | 0.000 | 0.000 |
| H471 | Shengdao14 | Jiaxiang, Shandong | 0.000 | 0.000 | 0.000 | 0.999 | 0.000 | 0.000 |
| H472 | Zhonghua3 | Haidian, Beijing | 0.000 | 0.000 | 0.000 | 0.000 | 0.000 | 1.000 |
| H473 | Zhongzuo93 | Tongzhou, Beijing | 0.000 | 0.000 | 0.000 | 0.000 | 0.999 | 0.000 |
| H474 | Jindao12 | Dongli, Tianjin | 0.000 | 0.000 | 0.000 | 0.999 | 0.000 | 0.000 |
| H475 | Jindao1007 | Dongli, Tianjin | 0.000 | 0.000 | 0.000 | 0.000 | 0.999 | 0.000 |
| H476 | Jinghui418 | Shenyang, Liaoning | 0.089 | 0.000 | 0.910 | 0.000 | 0.000 | 0.000 |
| H477 | Dongnongjing424 | Shenyang, Liaoning | 0.000 | 0.000 | 0.000 | 0.000 | 1.000 | 0.000 |
| H478 | Dongnongjingnuo418 | Shenyang, Liaoning | 0.000 | 0.000 | 0.000 | 0.000 | 0.999 | 0.000 |
| H479 | Shashani | Haerbin, Heilongjiang | 0.000 | 0.999 | 0.000 | 0.000 | 0.001 | 0.000 |
| H480 | Muzhan4 | Mudanjiang, Heilongjiang | 0.000 | 0.999 | 0.000 | 0.000 | 0.001 | 0.000 |
| H481 | Mudanjiang29 | Mudanjiang, Heilongjiang | 0.000 | 0.999 | 0.000 | 0.000 | 0.001 | 0.000 |
| H482 | Mudanjiang28 | Mudanjiang, Heilongjiang | 0.000 | 0.998 | 0.000 | 0.000 | 0.002 | 0.000 |
| H483 | Mudanjiang27 | Mudanjiang, Heilongjiang | 0.000 | 0.996 | 0.000 | 0.000 | 0.003 | 0.000 |
| H484 | Kenzhan2 | Nongken, Heilongjiang | 0.000 | 0.998 | 0.000 | 0.000 | 0.001 | 0.000 |
| H485 | Heijing8 | Haerbin, Heilongjiang | 0.000 | 0.998 | 0.000 | 0.000 | 0.002 | 0.000 |
| H486 | Hejing1 | Haerbin, Heilongjiang | 0.000 | 0.996 | 0.000 | 0.000 | 0.004 | 0.000 |
| H487 | Beidao4 | Haerbin, Heilongjiang | 0.000 | 0.999 | 0.000 | 0.000 | 0.001 | 0.000 |
| H488 | Beidao3 | Haerbin, Heilongjiang | 0.000 | 0.996 | 0.000 | 0.000 | 0.003 | 0.000 |
| H489 | Suijing12 | Suihua, Heilongjiang | 0.000 | 0.993 | 0.000 | 0.000 | 0.006 | 0.000 |
| H490 | Songjing12 | Songhuajiang, Heilongjiang | 0.000 | 0.996 | 0.000 | 0.000 | 0.003 | 0.000 |
| H491 | Songjing11 | Songhuajiang, Heilongjiang | 0.001 | 0.997 | 0.000 | 0.000 | 0.002 | 0.000 |
| H492 | Songjing10 | Songhuajiang, Heilongjiang | 0.000 | 1.000 | 0.000 | 0.000 | 0.000 | 0.000 |
| H493 | Dongnong430 | Haerbin, Heilongjiang | 0.000 | 0.999 | 0.000 | 0.000 | 0.000 | 0.000 |
| H494 | Dongnong424 | Haerbin, Heilongjiang | 0.000 | 1.000 | 0.000 | 0.000 | 0.000 | 0.000 |
| H495 | Longnuo3 | Haerbin, Heilongjiang | 0.000 | 1.000 | 0.000 | 0.000 | 0.000 | 0.000 |
| H496 | Longjing28 | Haerbin, Heilongjiang | 0.000 | 1.000 | 0.000 | 0.000 | 0.000 | 0.000 |
| H497 | Longjing27 | Haerbin, Heilongjiang | 0.000 | 1.000 | 0.000 | 0.000 | 0.000 | 0.000 |
| H498 | Longjing26 | Haerbin, Heilongjiang | 0.000 | 1.000 | 0.000 | 0.000 | 0.000 | 0.000 |
| H499 | Longjing25 | Haerbin, Heilongjiang | 0.000 | 1.000 | 0.000 | 0.000 | 0.000 | 0.000 |
| H500 | Longjing24 | Haerbin, Heilongjiang | 0.000 | 1.000 | 0.000 | 0.000 | 0.000 | 0.000 |
| H501 | Longjing22 | Haerbin, Heilongjiang | 0.000 | 1.000 | 0.000 | 0.000 | 0.000 | 0.000 |
| H502 | Longjing21 | Haerbin, Heilongjiang | 0.000 | 1.000 | 0.000 | 0.000 | 0.000 | 0.000 |
| H503 | Longjing20 | Haerbin, Heilongjiang | 0.000 | 1.000 | 0.000 | 0.000 | 0.000 | 0.000 |
| H504 | Longjing19 | Haerbin, Heilongjiang | 0.000 | 1.000 | 0.000 | 0.000 | 0.000 | 0.000 |
| H505 | Longjing18 | Haerbin, Heilongjiang | 0.000 | 0.999 | 0.000 | 0.000 | 0.000 | 0.000 |
| H506 | Longjing17 | Haerbin, Heilongjiang | 0.000 | 1.000 | 0.000 | 0.000 | 0.000 | 0.000 |
| H507 | Longjing16 | Haerbin, Heilongjiang | 0.000 | 1.000 | 0.000 | 0.000 | 0.000 | 0.000 |
| H508 | Longjing15 | Haerbin, Heilongjiang | 0.000 | 1.000 | 0.000 | 0.000 | 0.000 | 0.000 |
| H509 | Zhonglongdao1 | Haerbin, Heilongjiang | 0.000 | 1.000 | 0.000 | 0.000 | 0.000 | 0.000 |
| H510 | Longdao8 | Haerbin, Heilongjiang | 0.000 | 1.000 | 0.000 | 0.000 | 0.000 | 0.000 |
| H511 | Longdao6 | Haerbin, Heilongjiang | 0.000 | 1.000 | 0.000 | 0.000 | 0.000 | 0.000 |
| H512 | Longdao5 | Haerbin, Heilongjiang | 0.000 | 1.000 | 0.000 | 0.000 | 0.000 | 0.000 |
| H513 | Longdao4 | Haerbin, Heilongjiang | 0.000 | 1.000 | 0.000 | 0.000 | 0.000 | 0.000 |
| H514 | Kendao19 | Haerbin, Heilongjiang | 0.000 | 0.999 | 0.000 | 0.000 | 0.000 | 0.000 |
| H515 | Kendao18 | Haerbin, Heilongjiang | 0.000 | 1.000 | 0.000 | 0.000 | 0.000 | 0.000 |
| H516 | Kendao12 | Haerbin, Heilongjiang | 0.000 | 0.999 | 0.000 | 0.000 | 0.000 | 0.000 |
| H517 | Kendao13 | Haerbin, Heilongjiang | 0.000 | 0.999 | 0.000 | 0.000 | 0.000 | 0.000 |
| H518 | Kendao20 | Haerbin, Heilongjiang | 0.000 | 1.000 | 0.000 | 0.000 | 0.000 | 0.000 |
| H519 | Longdun106 | Haerbin, Heilongjiang | 0.000 | 1.000 | 0.000 | 0.000 | 0.000 | 0.000 |
| H520 | Longdun105 | Haerbin, Heilongjiang | 0.000 | 0.999 | 0.000 | 0.000 | 0.000 | 0.000 |
| H521 | Sanjiang2 | Haerbin, Heilongjiang | 0.000 | 1.000 | 0.000 | 0.000 | 0.000 | 0.000 |
| H522 | Nipponbare | Japan | 0.000 | 0.999 | 0.000 | 0.000 | 0.000 | 0.000 |
| H523 | Sasanishiki | Japan | 0.000 | 0.999 | 0.000 | 0.000 | 0.000 | 0.000 |
| H524 | M1004 | Japan | 0.000 | 1.000 | 0.000 | 0.000 | 0.000 | 0.000 |
| H525 | Chuugoku 91 | Japan | 0.000 | 0.999 | 0.000 | 0.000 | 0.000 | 0.000 |
| H526 | Akitakomachi | Japan | 0.000 | 1.000 | 0.000 | 0.000 | 0.000 | 0.000 |
| H527 | Akihikari | Japan | 0.000 | 1.000 | 0.000 | 0.000 | 0.000 | 0.000 |
| H528 | Koshihikari-R | Japan | 0.000 | 1.000 | 0.000 | 0.000 | 0.000 | 0.000 |
| H529 | Koshihikari-Q | Japan | 0.000 | 1.000 | 0.000 | 0.000 | 0.000 | 0.000 |
| H530 | Nishihikari | Japan | 0.000 | 0.999 | 0.000 | 0.000 | 0.000 | 0.000 |
| H531 | Koganebare | Japan | 0.000 | 0.999 | 0.000 | 0.000 | 0.000 | 0.000 |
| H532 | Aya | Japan | 0.000 | 0.999 | 0.000 | 0.000 | 0.000 | 0.000 |

**Supplementary Table 3.** Analysis of molecular variance (AMOVA) for the six subpopulations of rice accessions.

| Source of variation | d.f. | Sum of squares | Variance components | Percentage of variation | *P* value |
| --- | --- | --- | --- | --- | --- |
| Among subpopulations | 5 | 11128.90 | 14.17 | 28.93 | ＜0.001 |
| Among individuals within subpopulations | 468 | 32581.61 | 34.80 | 71.07 | ＜0.001 |
| Total | 473 | 43710.51 | 48.97 |  |  |

**Supplementary Table 4.** Comparison of Dʹ values for pairwise SSR loci in each subpopulation.

| Clusters | No.of LD^a^ locus pairs | Frequency of Dʹ^b^ value (*P*<0.05) | | | | | Mean of Dʹ |
| --- | --- | --- | --- | --- | --- | --- | --- |
|  |  | 0-0.2 | 0.2-0.4 | 0.4-0.6 | 0.6-0.8 | 0.8-1.0 |  |
| POP1 | 887 | 240 | 260 | 183 | 85 | 119 | 0.419 |
| POP2 | 1267 | 493 | 125 | 173 | 179 | 297 | 0.461 |
| POP3 | 1680 | 369 | 320 | 381 | 424 | 186 | 0.474 |
| POP4 | 1408 | 437 | 156 | 305 | 326 | 184 | 0.466 |
| POP5 | 1626 | 495 | 255 | 187 | 450 | 239 | 0.468 |
| POP6 | 1511 | 403 | 398 | 164 | 280 | 266 | 0.457 |

^a^ LD means linkage disequilibrium

^b^Dʹ means standardized disequilibrium coefficients

**Supplementary Table 5.** General linear correlation analysis for 4 grain traits.

|  | GL | GW | GT | TGW |
| --- | --- | --- | --- | --- |
| GL | - |  |  |  |
| GW | -0.66* | - |  |  |
| GT | -0.45* | 0.68* | - |  |
| TGW | 0.10 | 0.33* | 0.41* | - |

* Significant at *P*<0.01

**Supplementary Table 6.** Marker-trait associations with *P*-value less than 0.05, their equivalent false discovery rate probability (FDR), proportion of phenotypic variance explained (PVE), marker position on chromosome derived from 258 markers in six subpopulations.

| Traits | Subpopulations | Markers | Chr. | Position  /cM | 2010 | | |  | 2011 | | |  | 2012 | | |  | 2013 | | |
| --- | --- | --- | --- | --- | --- | --- | --- | --- | --- | --- | --- | --- | --- | --- | --- | --- | --- | --- | --- |
|  |  |  |  |  | *P* value | PVE | FDR |  | *P* value | PVE | FDR |  | *P* value | PVE | FDR |  | *P* value | PVE | FDR |
| GL | POP1 | RM6976 | 8 | 92.2 | 5.06E-03 | 0.211 | 7.14E-03 |  | 1.92E-02 | 0.167 | 3.00E-02 |  | 4.98E-03 | 0.211 | 7.14E-03 |  | 2.50E-02 | 0.229 | 2.50E-02 |
|  |  | RM209 | 11 | 84.7 | 4.23E-02 | 0.173 | 4.29E-02 |  | 2.72E-02 | 0.140 | 4.00E-02 |  | 4.01E-02 | 0.174 | 4.29E-02 |  | 3.28E-02 | 0.123 | 4.17E-02 |
|  | POP2 | RM21 | 11 | 85.7 | 2.20E-02 | 0.286 | 2.50E-02 |  | 1.08E-02 | 0.323 | 2.50E-02 |  | 1.73E-02 | 0.290 | 2.50E-02 |  | 3.94E-02 | 0.221 | 4.17E-02 |
|  | POP3 | RM3766 | 3 | 34.8 | 1.40E-03 | 0.163 | 9.68E-03 |  | 3.95E-03 | 0.144 | 1.54E-02 |  | 1.56E-03 | 0.161 | 1.67E-02 |  | 6.30E-03 | 0.135 | 1.67E-02 |
|  |  | RM335 | 4 | 5.4 | 2.30E-03 | 0.167 | 1.45E-02 |  | 6.82E-03 | 0.146 | 1.73E-02 |  | 2.17E-02 | 0.115 | 4.24E-02 |  | 2.20E-02 | 0.126 | 3.03E-02 |
|  |  | RM3836 | 4 | 108.2 | 1.45E-04 | 0.159 | 1.61E-03 |  | 7.66E-04 | 0.132 | 1.92E-03 |  | 1.25E-04 | 0.161 | 3.03E-03 |  | 1.23E-03 | 0.124 | 6.06E-03 |
|  |  | RM510 | 6 | 11.5 | 3.92E-03 | 0.159 | 2.10E-02 |  | 2.99E-03 | 0.164 | 1.35E-02 |  | 1.58E-02 | 0.131 | 3.79E-02 |  | 8.62E-04 | 0.190 | 3.03E-03 |
|  |  | RM276 | 6 | 33.5 | 7.01E-03 | 0.153 | 2.74E-02 |  | 2.23E-02 | 0.131 | 4.04E-02 |  | 3.07E-03 | 0.169 | 2.27E-02 |  | 9.58E-04 | 0.185 | 4.55E-03 |
|  |  | RM6976 | 8 | 92.2 | 6.72E-03 | 0.157 | 2.58E-02 |  | 1.65E-02 | 0.138 | 3.46E-02 |  | 2.79E-03 | 0.176 | 1.97E-02 |  | 3.91E-03 | 0.169 | 1.21E-02 |
|  |  | RM201 | 9 | 81.2 | 1.09E-02 | 0.126 | 3.23E-02 |  | 2.61E-02 | 0.109 | 4.23E-02 |  | 1.17E-02 | 0.124 | 3.48E-02 |  | 3.19E-02 | 0.104 | 4.09E-02 |
|  | POP4 | RM335 | 4 | 5.4 | 3.03E-02 | 0.202 | 3.57E-02 |  | 1.26E-02 | 0.243 | 2.50E-02 |  | 2.49E-02 | 0.210 | 4.00E-02 |  | 2.64E-02 | 0.199 | 4.03E-02 |
|  |  | RM348 | 4 | 160.8 | 7.33E-03 | 0.161 | 2.29E-02 |  | 3.96E-03 | 0.184 | 1.41E-02 |  | 4.32E-03 | 0.179 | 1.86E-02 |  | 5.96E-04 | 0.254 | 9.72E-03 |
|  |  | RM345 | 6 | 123.9 | 3.31E-03 | 0.231 | 1.00E-02 |  | 5.34E-03 | 0.212 | 1.72E-02 |  | 3.21E-04 | 0.325 | 5.71E-03 |  | 1.23E-02 | 0.179 | 2.92E-02 |
|  |  | RM125 | 7 | 24.8 | 3.67E-03 | 0.186 | 1.14E-02 |  | 1.52E-02 | 0.136 | 2.97E-02 |  | 8.58E-03 | 0.154 | 2.57E-02 |  | 9.25E-03 | 0.153 | 2.50E-02 |
|  |  | RM201 | 9 | 81.2 | 1.46E-02 | 0.269 | 2.86E-02 |  | 2.02E-02 | 0.261 | 3.59E-02 |  | 1.44E-02 | 0.269 | 3.43E-02 |  | 2.13E-02 | 0.223 | 3.61E-02 |
|  |  | RM167 | 11 | 37.5 | 1.40E-03 | 0.223 | 8.57E-03 |  | 1.11E-03 | 0.233 | 7.81E-03 |  | 3.25E-04 | 0.277 | 7.14E-03 |  | 2.09E-04 | 0.305 | 4.17E-03 |
|  | POP5 | RM525 | 2 | 143.7 | 1.40E-02 | 0.183 | 2.17E-02 |  | 2.16E-02 | 0.168 | 2.62E-02 |  | 1.94E-02 | 0.172 | 3.42E-02 |  | 2.81E-02 | 0.159 | 4.55E-02 |
|  |  | RM5639 | 3 | 39.8 | 1.85E-02 | 0.208 | 3.04E-02 |  | 2.42E-02 | 0.182 | 3.10E-02 |  | 1.88E-02 | 0.213 | 3.16E-02 |  | 7.36E-03 | 0.232 | 1.82E-02 |
|  |  | RM348 | 4 | 160.8 | 1.53E-03 | 0.128 | 4.35E-03 |  | 1.86E-03 | 0.143 | 2.38E-03 |  | 1.16E-03 | 0.135 | 7.89E-03 |  | 6.66E-03 | 0.092 | 1.59E-02 |
|  |  | RM1235 | 8 | 12.8 | 2.78E-03 | 0.182 | 8.70E-03 |  | 2.39E-03 | 0.187 | 7.14E-03 |  | 1.86E-03 | 0.195 | 1.32E-02 |  | 3.82E-03 | 0.173 | 1.14E-02 |
|  |  | RM6160 | 10 | 81 | 1.79E-02 | 0.101 | 2.83E-02 |  | 2.24E-02 | 0.095 | 2.86E-02 |  | 9.48E-03 | 0.118 | 2.37E-02 |  | 7.77E-03 | 0.123 | 2.05E-02 |
|  |  | RM6544 | 11 | 19.8 | 1.06E-02 | 0.082 | 1.96E-02 |  | 1.80E-02 | 0.070 | 2.14E-02 |  | 6.05E-03 | 0.095 | 2.11E-02 |  | 2.76E-02 | 0.089 | 4.32E-02 |
|  |  | RM209 | 11 | 84.7 | 1.76E-02 | 0.101 | 2.39E-02 |  | 3.68E-02 | 0.109 | 3.81E-02 |  | 1.69E-02 | 0.102 | 2.89E-02 |  | 1.75E-02 | 0.101 | 2.95E-02 |
|  |  | RM12 | 12 | 107.4 | 2.94E-03 | 0.150 | 1.30E-02 |  | 3.09E-03 | 0.148 | 9.52E-03 |  | 2.97E-03 | 0.149 | 1.84E-02 |  | 2.15E-03 | 0.159 | 6.82E-03 |
|  | POP6 | RM489 | 3 | 20.3 | 2.50E-02 | 0.216 | 3.68E-02 |  | 2.52E-02 | 0.216 | 2.62E-02 |  | 1.65E-02 | 0.234 | 1.71E-02 |  | 4.14E-02 | 0.190 | 4.57E-02 |
|  |  | RM3766 | 3 | 34.8 | 4.07E-02 | 0.147 | 4.47E-02 |  | 4.29E-02 | 0.142 | 4.64E-02 |  | 2.91E-02 | 0.159 | 3.66E-02 |  | 4.55E-02 | 0.138 | 4.57E-02 |
|  |  | RM276 | 6 | 33.5 | 2.48E-02 | 0.120 | 3.42E-02 |  | 2.85E-02 | 0.111 | 3.10E-02 |  | 1.23E-02 | 0.150 | 1.34E-02 |  | 1.29E-02 | 0.101 | 1.99E-02 |
|  |  | RM345 | 6 | 123.9 | 1.68E-03 | 0.302 | 5.26E-03 |  | 1.37E-03 | 0.311 | 4.76E-03 |  | 1.39E-03 | 0.310 | 2.44E-03 |  | 1.38E-02 | 0.215 | 1.43E-02 |
|  |  | RM6544 | 11 | 19.8 | 3.94E-02 | 0.155 | 4.21E-02 |  | 3.58E-02 | 0.156 | 3.81E-02 |  | 1.13E-02 | 0.218 | 1.22E-02 |  | 9.39E-03 | 0.180 | 1.14E-02 |
| GW | POP1 | RM5356 | 2 | 43.3 | 1.91E-02 | 0.172 | 3.21E-02 |  | 9.55E-03 | 0.179 | 2.00E-02 |  | 1.12E-02 | 0.205 | 2.22E-02 |  | 1.29E-02 | 0.191 | 2.69E-02 |
|  |  | RM317 | 4 | 96 | 2.05E-02 | 0.086 | 3.93E-02 |  | 1.47E-02 | 0.094 | 3.00E-02 |  | 8.46E-03 | 0.106 | 1.94E-02 |  | 1.00E-03 | 0.157 | 3.85E-03 |
|  |  | RM348 | 4 | 160.8 | 2.03E-02 | 0.059 | 3.57E-02 |  | 4.42E-02 | 0.043 | 4.50E-02 |  | 1.88E-02 | 0.060 | 2.78E-02 |  | 8.17E-03 | 0.154 | 1.54E-02 |
|  |  | RM528 | 6 | 100.8 | 3.94E-02 | 0.112 | 4.64E-02 |  | 6.99E-03 | 0.161 | 1.50E-02 |  | 2.11E-02 | 0.129 | 3.06E-02 |  | 1.07E-02 | 0.147 | 1.92E-02 |
|  |  | RM1019 | 8 | 0.5 | 5.99E-04 | 0.437 | 7.14E-03 |  | 3.35E-03 | 0.314 | 5.00E-03 |  | 1.09E-03 | 0.415 | 1.11E-02 |  | 3.40E-03 | 0.311 | 1.15E-02 |
|  | POP2 | RM6863 | 8 | 16.4 | 3.44E-02 | 0.135 | 5.00E-02 |  | 4.03E-02 | 0.205 | 5.00E-02 |  | 2.80E-02 | 0.229 | 5.00E-02 |  | 4.69E-02 | 0.238 | 5.00E-02 |
|  | POP3 | RM5356 | 2 | 43.3 | 1.11E-02 | 0.100 | 1.97E-02 |  | 4.79E-03 | 0.114 | 7.14E-03 |  | 3.21E-02 | 0.123 | 4.62E-02 |  | 3.53E-02 | 0.080 | 4.62E-02 |
|  |  | RM450 | 2 | 122.8 | 1.38E-03 | 0.106 | 5.26E-03 |  | 1.56E-03 | 0.105 | 4.76E-03 |  | 9.02E-03 | 0.079 | 2.69E-02 |  | 8.74E-03 | 0.079 | 2.12E-02 |
|  |  | RM3170 | 5 | 115.4 | 3.78E-04 | 0.174 | 2.63E-03 |  | 6.63E-04 | 0.164 | 2.38E-03 |  | 8.14E-03 | 0.118 | 2.31E-02 |  | 1.30E-02 | 0.111 | 2.69E-02 |
|  |  | RM1019 | 8 | 0.5 | 6.52E-04 | 0.194 | 3.95E-03 |  | 4.25E-04 | 0.203 | 1.19E-03 |  | 1.75E-03 | 0.172 | 3.85E-03 |  | 1.17E-02 | 0.138 | 2.50E-02 |
|  |  | RM6863 | 8 | 16.4 | 2.77E-03 | 0.139 | 7.89E-03 |  | 5.53E-03 | 0.127 | 1.19E-02 |  | 1.81E-02 | 0.103 | 3.08E-02 |  | 2.52E-06 | 0.263 | 1.92E-03 |
|  |  | RM247 | 12 | 26.7 | 2.86E-04 | 0.226 | 1.32E-03 |  | 7.70E-04 | 0.205 | 3.57E-03 |  | 5.71E-03 | 0.164 | 1.54E-02 |  | 2.13E-03 | 0.183 | 1.35E-02 |
|  | POP4 | RM525 | 2 | 143.7 | 2.48E-02 | 0.302 | 2.66E-02 |  | 3.77E-02 | 0.213 | 3.95E-02 |  | 7.19E-03 | 0.305 | 1.18E-02 |  | 1.22E-02 | 0.343 | 2.64E-02 |
|  |  | RM16 | 3 | 131.5 | 3.63E-03 | 0.191 | 1.09E-02 |  | 1.05E-02 | 0.153 | 1.84E-02 |  | 1.10E-02 | 0.150 | 1.76E-02 |  | 3.06E-03 | 0.198 | 1.53E-02 |
|  |  | RM348 | 4 | 160.8 | 1.05E-02 | 0.152 | 1.56E-02 |  | 1.25E-02 | 0.146 | 2.63E-02 |  | 3.59E-03 | 0.105 | 3.82E-02 |  | 3.82E-03 | 0.189 | 1.94E-02 |
|  |  | RM528 | 6 | 100.8 | 3.02E-02 | 0.143 | 3.28E-02 |  | 4.81E-03 | 0.139 | 1.05E-02 |  | 1.95E-02 | 0.171 | 2.35E-02 |  | 1.92E-02 | 0.116 | 3.61E-02 |
|  | POP5 | RM525 | 2 | 143.7 | 2.56E-02 | 0.162 | 2.60E-02 |  | 4.51E-02 | 0.142 | 4.72E-02 |  | 1.06E-02 | 0.168 | 1.47E-02 |  | 2.59E-02 | 0.117 | 2.61E-02 |
|  |  | RM348 | 4 | 160.8 | 1.13E-02 | 0.080 | 1.20E-02 |  | 3.63E-02 | 0.054 | 4.17E-02 |  | 1.98E-02 | 0.067 | 2.35E-02 |  | 1.52E-03 | 0.127 | 2.17E-03 |
|  |  | RM152 | 8 | 9.4 | 1.36E-02 | 0.228 | 1.40E-02 |  | 3.72E-02 | 0.189 | 3.89E-02 |  | 6.58E-03 | 0.256 | 8.82E-03 |  | 3.38E-02 | 0.191 | 3.91E-02 |
|  |  | RM6863 | 8 | 16.4 | 4.60E-02 | 0.121 | 4.80E-02 |  | 3.77E-02 | 0.127 | 4.44E-02 |  | 4.31E-02 | 0.147 | 4.41E-02 |  | 2.91E-02 | 0.113 | 3.04E-02 |
|  | POP6 | RM525 | 2 | 143.7 | 4.34E-02 | 0.212 | 4.47E-02 |  | 3.88E-02 | 0.223 | 4.69E-02 |  | 2.80E-03 | 0.249 | 1.67E-02 |  | 2.08E-02 | 0.237 | 2.50E-02 |
|  |  | RM277 | 12 | 48.2 | 1.05E-03 | 0.268 | 2.63E-03 |  | 1.73E-03 | 0.206 | 3.13E-03 |  | 2.18E-03 | 0.187 | 5.56E-03 |  | 4.38E-03 | 0.160 | 5.00E-03 |
| GT | POP1 | RM269 | 10 | 69.6 | 3.42E-02 | 0.110 | 4.00E-02 |  | 2.03E-02 | 0.125 | 2.14E-02 |  | 6.71E-03 | 0.111 | 1.33E-02 |  | 8.48E-03 | 0.133 | 1.25E-02 |
|  | POP2 | RM573 | 2 | 118.1 | 8.16E-03 | 0.289 | 1.67E-02 |  | 4.47E-02 | 0.200 | 5.00E-02 |  | 8.06E-03 | 0.293 | 1.25E-02 |  | 1.62E-02 | 0.251 | 1.67E-02 |
|  | POP3 | RM573 | 2 | 118.1 | 3.84E-02 | 0.100 | 3.85E-02 |  | 2.37E-02 | 0.127 | 2.50E-02 |  | 1.83E-02 | 0.113 | 2.00E-02 |  | 8.58E-05 | 0.171 | 4.55E-03 |
|  | POP4 | RM269 | 10 | 69.6 | 1.42E-03 | 0.315 | 5.56E-03 |  | 3.87E-03 | 0.269 | 4.55E-03 |  | 1.78E-02 | 0.182 | 1.88E-02 |  | 1.01E-02 | 0.294 | 1.54E-02 |
|  | POP5 | RM269 | 10 | 69.6 | 1.34E-03 | 0.081 | 5.56E-03 |  | 1.71E-02 | 0.126 | 1.82E-02 |  | 2.17E-02 | 0.147 | 4.29E-02 |  | 3.99E-02 | 0.079 | 4.00E-02 |
|  | POP6 | RM269 | 10 | 69.6 | 2.37E-03 | 0.139 | 1.25E-02 |  | 5.16E-03 | 0.136 | 1.00E-02 |  | 4.20E-02 | 0.142 | 4.50E-02 |  | 1.22E-02 | 0.171 | 1.67E-02 |
| TGW | POP1 | RM528 | 6 | 100.8 | 2.48E-02 | 0.126 | 2.73E-02 |  | 3.38E-02 | 0.137 | 3.89E-02 |  | 3.53E-02 | 0.116 | 3.64E-02 |  | 8.32E-03 | 0.139 | 2.00E-02 |
|  |  | RM345 | 6 | 123.9 | 3.06E-02 | 0.118 | 3.18E-02 |  | 2.38E-02 | 0.084 | 2.78E-02 |  | 1.18E-02 | 0.100 | 1.82E-02 |  | 3.91E-02 | 0.091 | 4.00E-02 |
|  |  | RM1019 | 8 | 0.5 | 7.19E-03 | 0.301 | 1.36E-02 |  | 8.70E-03 | 0.294 | 1.67E-02 |  | 9.68E-03 | 0.278 | 1.36E-02 |  | 1.28E-03 | 0.185 | 1.00E-02 |
|  |  | RM152 | 8 | 9.4 | 1.45E-03 | 0.149 | 4.55E-03 |  | 1.86E-03 | 0.142 | 5.56E-03 |  | 1.39E-03 | 0.150 | 4.55E-03 |  | 3.64E-03 | 0.072 | 3.00E-02 |
|  | POP2 | RM345 | 6 | 123.9 | 3.62E-03 | 0.289 | 2.00E-02 |  | 1.94E-02 | 0.183 | 2.86E-02 |  | 1.43E-02 | 0.219 | 3.75E-02 |  | 3.69E-02 | 0.085 | 4.39E-02 |
|  | POP3 | RM490 | 1 | 51 | 2.13E-02 | 0.088 | 2.67E-02 |  | 2.13E-02 | 0.089 | 2.33E-02 |  | 2.84E-03 | 0.097 | 3.13E-03 |  | 2.45E-02 | 0.096 | 2.50E-02 |
|  |  | RM168 | 3 | 122.8 | 1.89E-02 | 0.117 | 2.33E-02 |  | 3.71E-02 | 0.093 | 4.67E-02 |  | 3.01E-02 | 0.107 | 4.06E-02 |  | 2.72E-02 | 0.103 | 3.00E-02 |
|  |  | RM528 | 6 | 100.8 | 5.14E-03 | 0.127 | 6.67E-03 |  | 2.66E-02 | 0.097 | 3.33E-02 |  | 2.54E-02 | 0.097 | 3.44E-02 |  | 2.81E-02 | 0.098 | 3.50E-02 |
|  | POP4 | RM168 | 3 | 122.8 | 1.93E-02 | 0.224 | 2.50E-02 |  | 3.91E-02 | 0.317 | 5.00E-02 |  | 2.68E-02 | 0.188 | 3.33E-02 |  | 1.05E-03 | 0.291 | 7.14E-03 |
|  | POP5 | RM490 | 1 | 51 | 2.93E-02 | 0.125 | 3.57E-02 |  | 1.34E-02 | 0.119 | 2.86E-02 |  | 3.39E-02 | 0.078 | 4.17E-02 |  | 4.34E-02 | 0.102 | 4.55E-02 |
|  |  | RM1019 | 8 | 0.5 | 2.12E-02 | 0.097 | 2.86E-02 |  | 1.30E-02 | 0.088 | 2.14E-02 |  | 3.28E-02 | 0.118 | 3.33E-02 |  | 3.12E-02 | 0.082 | 3.64E-02 |
|  | POP6 | RM345 | 6 | 123.9 | 2.28E-02 | 0.163 | 2.50E-02 |  | 1.09E-02 | 0.179 | 2.50E-02 |  | 4.66E-03 | 0.174 | 1.25E-02 |  | 2.64E-02 | 0.136 | 2.86E-02 |

**Supplemental Table 7.** Positive elite alleles, phenotypic effect value and typical carrier materials for grain length, grain width, grain thickness and 1000-grain weight.

| Traits | Loci-alleles | Phenotypic effect value | | | | | Typical carrier materials |
| --- | --- | --- | --- | --- | --- | --- | --- |
|  |  | 2010 | 2011 | 2012 | 2013 | Mean |  |
| Grain length | RM3766-140 | 1.15 | 1.13 | 1.11 | 1.16 | 1.14 | Nongxiang 25 |
| (mm) | RM3766-130 | 1.19 | 1.18 | 1.16 | 1.25 | 1.20 | Yuedao 21 |
|  | RM335-160 | 0.57 | 0.56 | 0.62 | 0.68 | 0.61 | Yuedao 89 |
|  | RM335-155 | 1.48 | 1.42 | 1.45 | 1.44 | 1.45 | Yuedao 32 |
|  | RM348-135 | 1.34 | 1.29 | 1.40 | 1.46 | 1.37 | Yuedao 82 |
|  | RM276-140 | 1.12 | 1.16 | 1.18 | 1.16 | 1.16 | Yuzhenxiang |
|  | RM276-90 | 1.21 | 1.11 | 1.26 | 1.12 | 1.18 | Fengyouwan 8hao |
|  | RM276-100 | 1.32 | 1.20 | 1.39 | 1.18 | 1.27 | Yuedao 21 |
|  | RM345-150 | 0.45 | 0.47 | 0.50 | 0.55 | 0.49 | Wumangzaodao |
|  | RM345-165 | 1.04 | 1.01 | 1.11 | 1.16 | 1.08 | Yuedao 21 |
|  | RM345-175 | 1.22 | 1.20 | 1.24 | 1.21 | 1.22 | Yuedao 82 |
|  | RM345-170 | 1.22 | 1.26 | 1.25 | 1.23 | 1.24 | Fengyouwan 8hao |
|  | RM6976-155 | 0.52 | 0.55 | 0.54 | 0.62 | 0.56 | Yuedao 82 |
|  | RM6976-240 | 0.98 | 1.02 | 1.01 | 1.04 | 1.01 | Yuzhenxiang |
|  | RM201-160 | 0.93 | 0.95 | 0.94 | 1.03 | 0.96 | Yuedao 86 |
|  | RM201-165 | 1.19 | 1.15 | 1.25 | 1.15 | 1.19 | Nongxiang 25 |
|  | RM6544-145 | 0.92 | 0.88 | 0.97 | 0.94 | 0.93 | Yuedao 21 |
|  |  |  |  |  |  |  |  |
| Grain width | RM348-130 | 0.13 | 0.16 | 0.15 | 0.12 | 0.14 | Chushuhuang |
| (mm) | RM348-170 | 0.22 | 0.24 | 0.20 | 0.26 | 0.23 | Yangzhongdao |
|  | RM348-145 | 0.38 | 0.36 | 0.30 | 0.34 | 0.35 | Hongmangjing |
|  | RM528-185 | 0.18 | 0.16 | 0.20 | 0.23 | 0.19 | Cuyingwanyangdao |
|  | RM1019-160 | 0.14 | 0.18 | 0.17 | 0.15 | 0.16 | Chushuhuang |
|  | RM1019-120 | 0.16 | 0.20 | 0.17 | 0.21 | 0.19 | Yangzhongdao |
|  | RM1019-155 | 0.27 | 0.30 | 0.22 | 0.29 | 0.27 | Cuyingwanyangdao |
|  | RM1019-150 | 0.28 | 0.30 | 0.29 | 0.26 | 0.28 | Hongmangjing |
|  | RM6863-185 | 0.09 | 0.07 | 0.09 | 0.10 | 0.09 | Jiucaiqing |
|  | RM6863-190 | 0.14 | 0.18 | 0.14 | 0.13 | 0.15 | Shuangchengnuo |
|  |  |  |  |  |  |  |  |
| Grain thickness | RM573-185 | 0.02 | 0.01 | 0.01 | 0.02 | 0.02 | Yanjing 9hao |
| (mm) | RM573-235 | 0.03 | 0.04 | 0.03 | 0.05 | 0.04 | Cuyingwanyangdao |
|  | RM573-210 | 0.05 | 0.10 | 0.06 | 0.08 | 0.07 | Lianjing 2hao |
|  | RM573-220 | 0.06 | 0.07 | 0.09 | 0.08 | 0.08 | Si4263 |
|  | RM269-175 | 0.1 | 0.07 | 0.08 | 0.07 | 0.08 | Xudao 3hao |
|  | RM269-170 | 0.13 | 0.11 | 0.15 | 0.18 | 0.14 | Wanhuangdao |
|  |  |  |  |  |  |  |  |
| 1,000-grain weight | RM490-105 | 0.23 | 0.27 | 0.20 | 0.22 | 0.23 | Maozitou |
| (g) | RM490-100 | 0.32 | 0.29 | 0.26 | 0.27 | 0.28 | Yuedao 86 |
|  | RM528-245 | 0.42 | 0.40 | 0.37 | 0.45 | 0.41 | 9311 |
|  | RM528-185 | 0.66 | 0.69 | 0.67 | 0.65 | 0.67 | Xiangjing9407 |
|  | RM345-160 | 0.26 | 0.27 | 0.24 | 0.23 | 0.25 | Si4033 |
|  | RM345-105 | 0.32 | 0.28 | 0.29 | 0.26 | 0.29 | Maozitou |
|  | RM345-155 | 0.36 | 0.32 | 0.39 | 0.37 | 0.36 | Yuedao 86 |
|  | RM345-150 | 0.58 | 0.52 | 0.55 | 0.57 | 0.55 | Xiangjing9407 |

**SupplementaryTable 8.** Parental combinations and numbers of elite alleles after combinations predicted from association mapping of grain traits.

| Traits | Parental combinations | No. of elite alleles predicted |
| --- | --- | --- |
| Grain length | Yuzhenxiang × Yuedao 82 | 7 |
|  | Yuzhenxiang × Yuedao 21 | 8 |
|  | Yuedao 21 × Nongxiang 25 | 8 |
|  | Yuedao 82 × Yuedao 21 | 8 |
|  | Yuedao 21 × Fengyouwan 8hao | 8 |
|  |  |  |
| Grain width | Shuangchengnuo × Hongmangjing | 3 |
|  | Yangzhongdao ×Cuyingwanyangdao | 4 |
|  | Hongmangjing × Cuyingwanyangdao | 4 |
|  | Cushuhuang ×Cuyingwanyangdao | 4 |
|  | Shuangchengnuo × Cuyingwanyangdao | 4 |
|  |  |  |
| Grain thickness | Xudao 3hao× Wanhuangdao | 2 |
|  | Si4263 ×Wanhuangdao | 2 |
|  | Si4263 × Cuganhuangdao | 2 |
|  | Si4263 × Xudao 3hao | 2 |
|  | Si4263 × Lianjing 2hao | 2 |
|  |  |  |
| 1,000-grain weight | Yuedao 86 × 9311 | 3 |
|  | Xiangjing9407 × 9311 | 3 |
|  | Xiangjing9407 × Si4033 | 3 |
|  | Xiangjing9407 × Maozitou | 3 |
|  | Xiangjing9407 ×Yuedao 86 | 3 |

**Supplemental Table 9. Elite alleles carried by the superior parents for grain traits and corresponding phenotypic effect.**

| Traits | Superior parents | Locus-allele (corresponding phenotypic effect value) |
| --- | --- | --- |
| Grain length | Yuzhenxiang | RM3766-140(1.14), RM276-140(1.16), RM345-170(1.24), RM6976-240(1.01), RM201-165(1.19) |
|  | Nongxiang 25 | RM3766-140(1.14), RM276-90(1.18), RM345-170(1.24), RM6976-240(1.01), RM201-165(1.19) |
|  | Yuedao 82 | RM3766-130(1.20), RM335-160(0.61), RM345-175(0.56), RM6976-155(0.56), RM201-160(0.96), RM6544-145(0.93) |
|  | Yuedao 21 | RM3766-130(1.20), RM335-155(1.45), RM348-135(1.37), RM276-100(1.27), RM345-165(1.08), RM201-165(1.19), RM6544-145(0.93) |
|  | Fengyouwan 8hao | RM276-90(1.18), RM345-170(1.24), RM6976-240(1.01) |
| Grain width | Shuangchengnuo | RM348-145(0.35), RM6863-190(0.15) |
|  | Cuyingwanyangdao | RM528-185(0.19), RM1019-155(0.27), RM6863-190(0.15) |
|  | Yangzhongdao | RM348-170(0.23), RM1019-120(0.19) |
|  | Chushuhuang | RM348-145(0.35), RM1019-160(0.16), RM6863-190(0.15) |
|  | Hongmangjing | RM348-145(0.35), RM1019-150(0.28) |
| Grain thickness | Cuganhuangdao | RM573-235(0.04) |
|  | Si4263 | RM573-220(0.08), RM269-170(0.14) |
|  | Wanhuangdao | RM269-170(0.14) |
|  | Xudao 3hao | RM573-210(0.07), RM269-175(0.08) |
|  | Lianjing 2hao | RM573-210(0.07), RM269-170(0.14) |
| 1,000-grain weight | Yuedao 86 | RM490-100(0.28), RM345-155(0.36) |
|  | Si4033 | RM528-245(0.41), RM345-160(0.25) |
|  | Xiangjing9407 | RM490-100(0.28), RM528-185(0.67), RM345-150(0.55) |
|  | Maozitou | RM490-105(0.23), RM345-105(0.29) |
|  | 9311 | RM528-245(0.41) |

**Supplementary Table 10.** The list for QTLs identified from this study and shared in previous studies.

| Traits | SSR  markers | Chromosome | Start position  /bp^a^ | End position  /bp^a^ | QTL reported in the previous studies | | |
| --- | --- | --- | --- | --- | --- | --- | --- |
|  |  |  |  |  | Start position  /bp^a^ | End position /bp^a^ | Reference |
| Grain length | RM3766 | 3 | 6,933,576 | 6,933,717 | 8,320,001 | 13,221,482 | Aluko et al. (2004) |
|  | RM348 | 4 | 32,650,358 | 32,650,358 | 31,065,233 | 34,698,199 | Redoňa and Mackill (1998) |
|  | RM345 | 6 | 30, 864,845 | 30,864,999 | 24,035,491 | 31,206,401 | Aluko et al. (2004) |
|  | RM6976 | 8 | 23,555,534 | 23,555,817 | 21,427,234 | 26,700,842 | Agrama et al. (2007) |
| Grain width | RM6863 | 8 | 2,011,124 | 2,011,521 | 1,527,393 | 4,106,001 | Redoňa and Mackill (1998) |
| Grain thickness | RM269 | 10 | 17,817,336 | 17,817,657 | 13,926,405 | 22,323,258 | Li et al. (2004) |
|  |  |  |  |  | 16,691,782 | 17,934,502 | Bai et al. (2010) |
| 1,000-grain weight | RM490 | 1 | 13,056,089 | 13,056,247 | 10,265,600 | 15,008,029 | Lu et al. (1996) |
|  |  |  |  |  | 4,753,787 | 16,486,442 | Hua et al. (2002); Xing et al. (2002) |
|  |  |  |  |  | 7,270,403 | 16,486,411 | Lin et al. (1996) |
|  | RM528 | 6 | 26,554,756 | 26,554,987 | 24,916,395 | 28,640,541 | Hua et al. (2002) |
|  |  |  |  |  | 23,348,366 | 27,659,063 | Ishimaru et al. (2003) |
|  | RM345 | 6 | 30,864,845 | 30,864,999 | 17,792,102 | 32,691,241 | Xing et al. (2002) |

^a^ The estimated physical position (bp) was inferred the Gremene (http://www.gramene.org/markers) and NCBI (http://blast.ncbinlm.nih.gov/Blast.cgi)


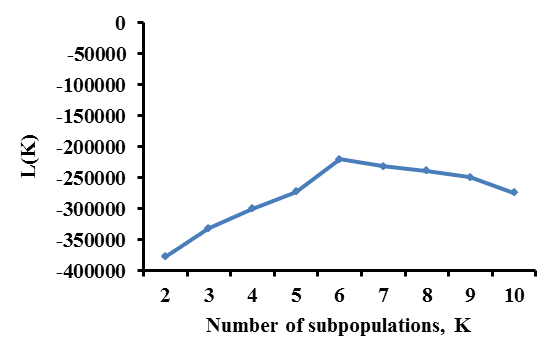


**Supplementary Fig. 1.** Log probability of data, L(K), averaged over the replicates.


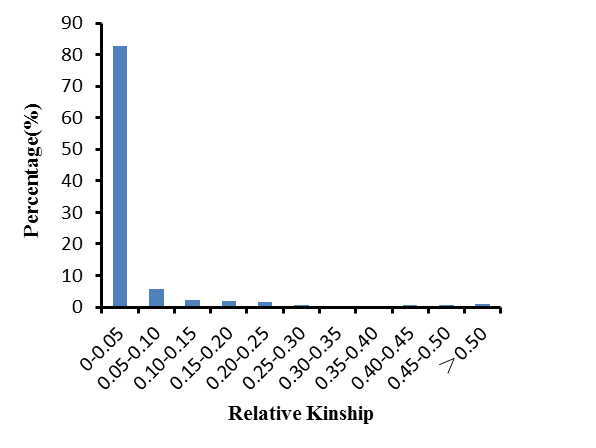


**Supplementary Fig. 2.** Distribution of pair-wise kinship coefficients among 532 rice accessions. Kinship coefficients between accessions were calculated using 258 SSR markers.
